# BHLHE40 Inhibits Ferroptosis in Pancreatic Cancer Cells via Upregulating SREBF1

**DOI:** 10.1002/advs.202306298

**Published:** 2023-12-08

**Authors:** Yizhi Cao, Xuelong Wang, Yang Liu, Pengyi Liu, Jiejie Qin, Youwei Zhu, Shuyu Zhai, Yongsheng Jiang, Yihao Liu, Lijie Han, Jiaxin Luo, Ronghao Zhang, Minmin Shi, Liwen Wang, Xiaomei Tang, Meilin Xue, Jia Liu, Weishen Wang, Chenlei Wen, Xiaxing Deng, Chenghong Peng, Hao Chen, Dongfeng Cheng, Lingxi Jiang, Baiyong Shen

**Affiliations:** ^1^ Department of General Surgery Pancreatic Disease Center Ruijin Hospital Shanghai Jiaotong University School of Medicine Shanghai 200025 P. R. China; ^2^ Research Institute of Pancreatic Diseases Shanghai Key Laboratory of Translational Research for Pancreatic Neoplasms Shanghai Jiaotong University School of Medicine Shanghai 200025 P. R. China; ^3^ State Key Laboratory of Oncogenes and Related Genes Institute of Translational Medicine Shanghai Jiaotong University Shanghai 200025 P. R. China; ^4^ Institute of Translational Medicine Shanghai Jiaotong University Shanghai 200025 P. R. China; ^5^ Shanghai Key Laboratory of Pancreatic Neoplasms Translational Medicine Shanghai 200025 P. R. China

**Keywords:** BHLHE40, ferroptosis, lipid peroxidation, pancreatic cancer, SREBF1

## Abstract

Pancreatic cancer (PCa) is one of the most fatal human malignancies. The enhanced infiltration of stromal tissue into the PCa tumor microenvironment limits the identification of key tumor‐specific transcription factors and epigenomic abnormalities in malignant epithelial cells. Integrated transcriptome and epigenetic multiomics analyses of the paired PCa organoids indicate that the basic helix‐loop‐helix transcription factor 40 (BHLHE40) is significantly upregulated in tumor samples. Increased chromatin accessibility at the promoter region and enhanced mTOR pathway activity contribute to the elevated expression of BHLHE40. Integrated analysis of chromatin immunoprecipitation‐seq, RNA‐seq, and high‐throughput chromosome conformation capture data, together with chromosome conformation capture assays, indicate that BHLHE40 not only regulates sterol regulatory element‐binding factor 1 (SREBF1) transcription as a classic transcription factor but also links the enhancer and promoter regions of SREBF1. It is found that the BHLHE40‐SREBF1‐stearoyl‐CoA desaturase axis protects PCa cells from ferroptosis, resulting in the reduced accumulation of lipid peroxidation. Moreover, fatostatin, an SREBF1 inhibitor, significantly suppresses the growth of PCa tumors with high expressions of BHLHE40. This study highlights the important roles of BHLHE40‐mediated lipid peroxidation in inducing ferroptosis in PCa cells and provides a novel mechanism underlying SREBF1 overexpression in PCa.

## Graphical abstract

This study investigates the role of basic helix‐loop‐helix transcription factor 40 (BHLHE40) in pancreatic cancer (PCa), revealing its upregulation in tumor due to increased chromatin accessibility and mTOR pathway activity. BHLHE40 regulates sterol regulatory element‐binding factor 1 (SREBF1) transcription and links enhancer and promoter regions, impacting lipid peroxidation and ferroptosis. The findings suggest that inhibiting SREBF1 with fatostatin could suppress PCa tumor growth, especially in cases with high BHLHE40 expression.

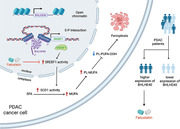

## 1 Introduction

Cancer develops due to genetic abnormalities that take place in somatic cells, resulting in dysregulated transcriptional networks. “Transcriptional addiction” refers to the behavior of cancer cells that exhibit high dependence on certain oncogenic regulators acquired during tumor development and that remain critical for tumor progression.^[^ ^1^ ^]^ Pancreatic cancer (PCa) is one of the deadliest types of cancer with a 5 year survival rate of 9%.^[^ ^2^ ^]^ Key somatic mutations and master regulators of transcription programs have been identified via high‐throughput sequencing.^[^ ^3^ ^]^ Although certain molecular subtypes have been characterized, tumor‐specific transcription factors and epigenetic abnormalities need to be explored because of the strong heterogeneity of PCa.

PCa tissues exhibit an extensive stromal component that comprises 50%–80%^[^ ^4^ ^]^ of the overall pancreatic tumor microenvironment. This reduction in the neoplastic cellularity of PCa makes it challenging to isolate a sufficient number of epithelium‐derived malignant cells for high‐throughput sequencing. In particular, precise measurements of tumor‐specific features at the epigenomic level are limited because >10^6^ cells are required for the genome‐wide profiling of chromatin accessibility regions and the mapping of regions of DNA‐associated protein interactions.^[^ ^1^ ^]^ Pancreatic organoids present a solution to this challenge, because PCa‐derived organoids not only recapitulate physiologically relevant components of in vitro tumor progression but also provide an adequate number of malignant cells for epigenomic sequencing.^[^ ^5^ ^]^ An integrated analysis of the assay for transposase‐accessible chromatin using sequencing (ATAC‐seq), cleavage under targets and tagmentation (CUT&Tag) profiling of the H3K27ac modification of PCa paired organoids, and transcriptome data of PCa from The Cancer Genome Atlas (TCGA) has identified several potential master transcription factors, of which the basic helix‐loop‐helix transcription factor 40 (BHLHE40) is of interest because it ranks at the top of the list.

BHLHE40 is a known immune response mediator involved in infection, autoimmunity, and inflammation.^[^ ^6^ ^]^ We previously reported that BHLHE40 is associated with hypoxia‐related stress and glucose metabolism in PCa tumor‐associated neutrophils.^[^ ^7^ ^]^ However, the roles and molecular mechanisms of BHLHE40 in PCa tumor cells have not yet been characterized. Notably, we found that BHLHE40 contained large intrinsically disordered regions (IDRs) that could multivalently interact with each other, enabling the formation of phase‐separated droplets. In recent years, phase‐separated condensates have been reported to facilitate a structured genome.^[^ ^8^, ^9^ ^]^ Furthermore, an integrated analysis of RNA‐seq, ChIP‐seq, and public high‐throughput chromosome conformation capture (Hi‐C) data implied that BHLHE40 upregulates the expression of sterol regulatory element‐binding protein (SREBF1, encoding SREBP) via linking its promoter‐enhancer regions. SREBF1 is a master regulator of fatty acid metabolism that is often over‐expressed in cancers.^[^ ^10^ ^]^ Dysregulated lipid metabolism is one of the most prominent metabolic alterations in PCa cells compared to normal ductal cells^[^ ^11^ ^]^; therefore, unveiling the role of BHLHE40‐SREBF1 in PCa progression is important.

Lipogenesis increases the degree of lipid saturation while decreasing the relative degree of polyunsaturation, thereby protecting cancer cells from oxidative‐induced cell death.^[^ ^12^ ^]^ In early life, polyunsaturated fatty acids (PUFAs) were involved in lipid metabolism and membrane biochemistry, which increase the tunability of membrane fluidity.^[^ ^13^ ^]^ After the Great Oxygenation Event, the presence of PUFAs caused the cell membranes to become a source of damaging oxidative reactions as the lipid peroxides were generated, and this peroxidation reaction was markedly accelerated by Fe(II).^[^ ^14^ ^]^ The accumulation of lipid‐based reactive oxygen species (ROS) resulted in ferroptosis.^[^ ^15^ ^]^ The distinctive metabolism of cancer cells, accumulation of ROS, and specific mutations in RAS family members, such as HRAS and KRAS, render cancer cells intrinsically susceptible to ferroptosis, thus exhibiting vulnerabilities that could be therapeutically targeted.^[^ ^16^ ^]^ Conventional cancer therapies, including chemotherapy, radiotherapy, and immunotherapy, can trigger ferroptosis.^[^ ^17^, ^18^ ^]^ Therefore, ferroptosis inducers have significant potential in cancer therapy, particularly when used in combination with conventional therapies.^[^ ^18^, ^19^ ^]^

Here, integrated transcriptome and epigenetic multi‐omics analyses of paired (tumor and para‐tumor tissues) PCa organoids indicated that BHLHE40 was upregulated in PCa. Moreover, the upregulation of BHLHE40 was associated with the activity of the mTOR pathway and chromatin accessibility at the promoter region. As a transcription factor, BHLHE40 not only promoted the transcription of SREBF1, but also facilitated the linking of the promoter‐to‐enhancer regions of SREBF1 by forming phase‐separated condensates. SREBF1 reprogrammed fatty acid metabolism in PCa by regulating downstream targets (FASN, ACLY, and SCD1) and inhibiting ROS‐induced ferroptosis. Thus, the SREBF1 inhibitor, fatostatin, significantly decreased the growth of PCa tumors with high BHLHE40 expression by inducing ferroptosis.

## 2 Results

### 2.1 Open Chromatin at the BHLHE40 Locus in PCa Patient‐Derived Organoids

**Figure 1:**
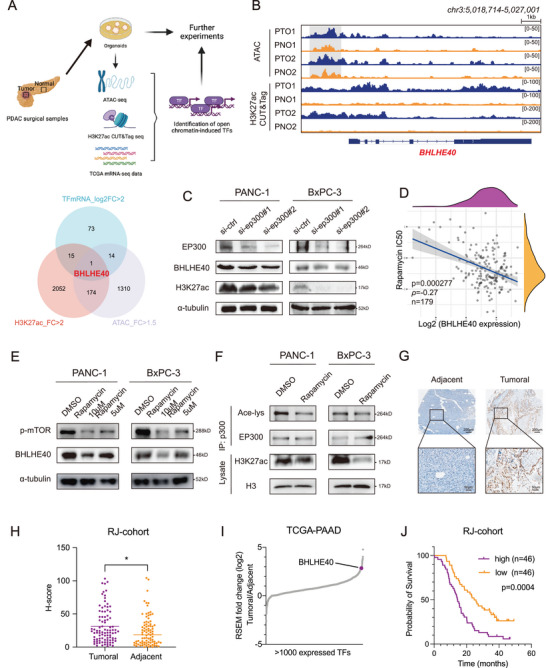
Identification of BHLHE40 in pancreatic ductal adenocarcinoma and its upstream regulators. A) UP: Illustrations of the identification strategies of transcription factors. DOWN: Venn diagram of intersected transcription factors with H3K27ac and chromatin openness gain peaks and elevated mRNA expression. B) Integrative Genomics Viewer plots showing ATAC‐ and ChIP‐seq profiles of indicated changes of chromatin openness or H3K27ac enrichment in the BHLHE40 gene locus. C) Protein levels of BHLHE40 and H3K27ac significantly decreased after the EP300 knockdown. D) Negative correlation between the expression of BHLHE40 and IC_50_ score of rapamycin was confirmed by Spearman correlation analysis. The ordinate shows the distribution of the IC_50_ score, and the abscissa shows the various expression levels of the samples (*P* = 0.000277, *ρ* = −0.27, *n* = 179). E) Rapamycin, an mTOR inhibitor, was selected to treat PANC‐1 and BxPC‐3 cell lines, and a significant decrease in phosphorylated mTOR and BHLHE40 levels was observed in a dose‐dependent manner. F) Acetylation of EP300 and histone H3 in PANC and BxPC‐3 cells treated with or without mTORC1 inhibitor (Rapamycin at 10 × 10^−6^ m). Immunoprecipitated EP300 and lysate histone H3 were analyzed using western blotting with anti‐acetyl‐lysine and anti‐acetyl‐histone H3 antibodies, respectively. G) Immunohistochemistry assay validated that the levels of BHLHE40 were higher in tumoral tissues than in adjacent tissues. Scale bar, 200 µm (upper), 50 µm (bottom). H) Comparison of H‐scores between tumoral and adjacent tissues for BHLHE40 immunohistochemistry staining. I) Expressed transcriptional factors ranked by mean log2 fold‐change in primary PCa tumoral versus adjacent tissues in TCGA‐PAAD datasets. BHLHE40 was labeled and ranked in the front. J) Kaplan–Meier analysis in the RJ‐1 dataset suggested that patients with high BHLHE40 levels experienced worse overall survival outcomes than those with low BHLHE40 levels (*P* = 0.0004, *n* = 92). Data presentation: H) Data were the mean ± s.d. of *n* = 84 samples for each group. Statistical analysis: spearman rank correlation test for (D); paired two‐sided t‐test for (H); log‐rank test for (J). Source data are provided as described in availability of data and materials section. *P < 0.05.

To explore the epigenomic abnormalities in PCa tumorigenesis, two pairs of tumoral (PTO1 and PTO2) and para‐tumoral (PNO1 and PNO2) tissue organoids were analyzed using ATAC‐seq and CUT&Tag H3K27ac modification profiling (**Figure** **1**A). H&E staining and whole‐exome sequencing were performed to confirm the morphological and genetic characteristics of the tumoral and para‐tumoral organoids (Figure S1A,B, Supporting Information). By evaluating the intersection of genes in groups of differentially expressed transcription factors from mRNA‐seq of the PAAD dataset in TCGA, H3K27ac CUT&Tag analysis of the enhanced binding sites (Figure S1C,D, Supporting Information), and ATAC‐seq of upregulated accessibility‐enhanced regions (Figure S1E, Supporting Information) in PCa organoids, a transcription factor (BHLHE40) was identified for further investigation (Figure 1A, Table S1, Supporting Information). Compared to the PNOs, H3K27ac was enriched in the promoter region of BHLHE40 in PCa organoids (Figure 1B). The EP300/CPB complex is the primary modulator of H3K27ac modifications in chromatin.^[^ ^20^ ^]^ TCGA‐PAAD and GTEx data demonstrated that the mRNA level of BHLHE40 was significantly positively associated with that of EP300 (Figure S2A, Supporting Information). EP300 silencing or inhibition (using C646 as an inhibitor) consistently decreased the H3K27ac expression level of BHLHE40 in PANC‐1 and BxPC‐3 cell lines (Figure 1C and Figure S2B,C, Supporting Information). Thus, we concluded that H3K27ac modification mediated by EP300 contributed to the upregulation of BHLHE40. As the induction of BHLHE40 in hepatocytes could be prevented by the inhibitor of mTOR signaling, rapamycin,^[^ ^21^ ^]^ we subsequently investigated the effects of mTOR signaling on the expression of BHLHE40. Clinical samples or PCa cells with higher BHLHE40 expressions had lower rapamycin IC_50_ values, suggesting that these samples were more vulnerable to rapamycin (Figure 1D and Figure S2D–I, Supporting Information). Rapamycin decreased the expression levels of BHLHE40 in PANC‐1 and BxPC‐3 cells (Figure 1E). A previous study has shown that mTOR activates EP300 to induce lipogenesis.^[^ ^22^ ^]^ To confirm the regulatory role of mTOR‐EP300 in BHLHE40 expression, we further demonstrated that rapamycin treatment significantly reduced the activation of EP300 both in PANC‐1 and BxPC‐3 cells (Figure 1F and Figure S2J, Supporting Information). In addition, the protein expressions of EP300, phosphorylated mTOR, and BHLHE40 were positively correlated across PCa cell lines (Figure S2N, Supporting Information). Compared with other cancer types, the mRNA level of BHLHE40 was upregulated in PCa (Figure S2K, Supporting Information). The IHC staining of PCa clinical samples showed that BHLHE40 was expressed at a higher level in tumor samples than in para‐tumor tissues (Figure 1G,H). Similar results at the RNA level were observed for the GEO (GSE16515) and TCGA‐PAAD datasets (Figure 1I and Figure S2L, Supporting Information). Higher BHLHE40 expression was significantly associated with reduced overall survival in patients with PCa (Figure 1J and Figure S2M, Supporting Information). In summary, increased chromatin accessibility at the promoter region and enhanced mTOR pathway activity contribute to the elevated expression of BHLHE40, which renders an unfavorable prognosis in PCa.

### 2.2 Knockdown of BHLHE40 Decreases the Tumorigenesis and Metastasis of PCa

**Figure 2:**
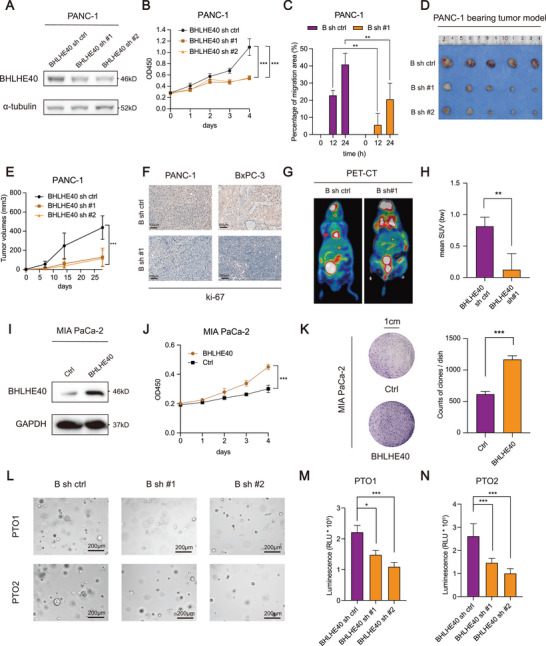
BHLHE40 promotes PCa proliferation and liver metastases in vitro and in vivo. A) Western blot of BHLHE40 knockdown efficiency in PANC‐1 cell lines. B,C) Effects of BHLHE40 knockdown on the proliferation and migration ability of the PANC‐1 cell lines were measured using CCK‐8 and wound‐healing assays. D) PCa, PANC‐1, and BxPC‐3 (gross anatomy also shown in Figure S3, Supporting Information) cells stably transfected with BHLHE40 sh ctrl or sh #1 were injected subcutaneously into 4 week old BALB/c male nude mice (*n* = 5 for each group). Representative images are of dissected xenogeneic tumors from nude mice in each group. E) Tumor volumes were measured and calculated after subcutaneous injection. F) Tumor tissues were subjected to Ki‐67 staining. Scale bar, 100 µm. G) Representative micro‐PET/CT image of the metastatic xenograft mouse model. H) BHLHE40 knockdown decreased the mean SUV value of metastatic liver tumors, reinforcing the role of BHLHE40 in promoting metastasis in vivo. I) Western blot of BHLHE40 overexpression efficiency in MIA PaCa‐2 cell lines. J,K) Effects of BHLHE40 overexpression on the proliferation ability of the MIA PaCa‐2 cell line was measured using the CCK‐8 and colony formation assays. L–N) Representative pictures of two PCa organoids (PTO1 and PTO2) transfected with BHLHE40 knockdown vectors or control lentivirus for 10 d (scale bar, 200 µm, left panel) and quantified via organoid diameters (right panel). PET, positron emission tomography; mean SUV, mean standardized uptake value; "B sh" or “BHLHE40 sh,” short for BHLHE40 short hairpin RNA. Data presentation: B,E,J,M,N) data were the mean ± s.d. of *n* = 6 independent experiments; E) data were the mean ± s.d. of *n* = 5 independent experiments; H) data were the mean ± s.d. of *n* = 4 independent experiments; C,K) Data were the mean ± s.d. of *n* = 3 independent experiments. Statistical analysis: two‐way ANOVA for (B,E,J); unpaired two‐sided t‐test for (C,H,K,M,N). * *P* < 0.05, ** *P* < 0.01, *** *P* < 0.001.

To explore the functional roles of BHLHE40 in PCa, BHLHE40 was silenced in two cell lines (PANC‐1 and BxPC‐3) with high expressions and overexpressed in MIA PaCa‐2 cells (Figure S2N, Supporting Information). The knockdown and overexpression of BHLHE40 were evaluated using western blotting (**Figure** **2**A and Figure S3A, Supporting Information). BHLHE40 knockdown decreased cell proliferation (Figure 2B and Figure S3B–D, Supporting Information) and migration (Figure 2C and Figure S3E–J, Supporting Information). Subcutaneous xenograft experiments indicated that the tumors induced by BHLHE40‐silencing cells were significantly smaller than those induced by controls (Figure 2D–E and Figure S3K–N, Supporting Information). IHC staining indicated that Ki‐67 abundance was reduced in the BHLHE40 knockdown group (Figure 2F). Furthermore, PET/CT luminescence signals showed that BHLHE40 silencing partially inhibited the metastatic ability of PCa cells in the liver (Figure 2G,H and Figure S4A,B, Supporting Information). BHLHE40 overexpression enhanced the in vitro and in vivo tumorigenicity of PCa cells (Figure 2I–K). Similarly, BHLHE40 knockdown in two established PCa organoids (PTO1 and PTO2) reduced cell proliferation (Figure 2L–N). Therefore, these data suggest that BHLHE40 has a strong oncogenic function.

### 2.3 BHLHE40 Regulates Key Factors of Fatty Acid Metabolism in PCa via SREBF1

**Figure 3:**
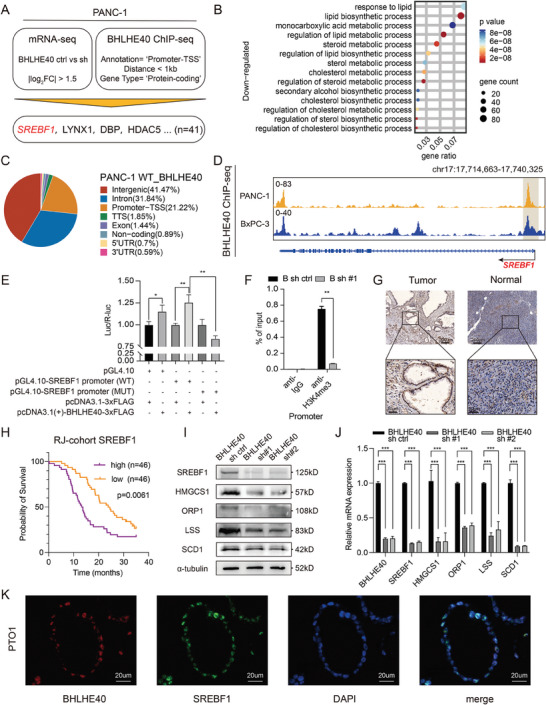
Overexpression of BHLHE40 promotes tumorigenesis via altering the oncogenic transcriptome in PCa cells. A) Integrated analysis flowchart of RNA‐ and ChIP‐seq in PANC‐1 cells. Downregulated genes after BHLHE40 knockdown with log2 fold change >1.5 times were intersected with binding loci of BHLHE40. B) Bubble plot showing GO enrichment analysis of downregulated genes related to fatty acid metabolism after BHLHE40 knockdown. C) Pie chart of the distributions of BHLHE40‐binding regions. D) Close view of the SREBF1 gene loci and BHLHE40 ChIP‐seq signal in PANC‐1 and BxPC‐3 cells. E) Luciferase reporter assay results showed that BHLHE40 overexpression promoted the luciferase activity of the pGL4.10‐SREBF1 promoter (WT) rather than the mutant promoter. F) Enrichment levels of H3K4me3 at the promoter region of SREBF1 in BHLHE40 knockdown or control PANC‐1 cell were determined via ChIP‐qPCR assay. G) Representative images of SREBF1 IHC staining of PCa tumor and para‐tumor tissues. Scale bar, 200 µm (upper) or 50 µm (bottom). H) High SREBF1 protein levels also correlated with reduced overall survival (OS) outcomes based on the analysis of tissue microarray data from the RJ‐1 dataset. I,J) Expression levels of SREBF1 and subsequent fatty acid metabolism related proteins (HMGCS1, ORP1, LSS, and SCD1) were measured using western blot and qPCR assays in BHLHE40 knockdown or control PANC‐1 cells. K) Representative images of double immunofluorescence staining of SREBF1 (green) and BHLHE40 (red) in PTO1. DAPI (blue) was used for nuclei counterstaining. Data presentation: E,F,J) data were the mean ± s.d. of *n* = 3 independent experiments. Statistical analysis: one‐sided Fisher's exact test for (B); unpaired two‐sided t‐test for E,F,J); log‐rank test for (H); *P < 0.05, **P < 0.01, ***P < 0.001.

To investigate the underlying mechanism of action of BHLHE40 in PCa progression, we conducted transcriptome profiling of BHLHE40‐silencing PCa cell lines (**Figure** **3**A). Transcriptome analysis revealed 2712 DEGs between the BHLHE40 knockdown and control groups, with 1595 upregulated and 1117 downregulated genes (Figure S5A, Supporting Information). GO analysis suggested that DEGs were significantly enriched in cancer‐related pathways, such as the regulation of cell migration (Figure S5B, Supporting Information). Furthermore, DEGs were enriched in the biological process of fatty acid metabolism (Figure 3B). Similar results were observed in the KEGG enrichment analysis (Figure S5C, Supporting Information). ChIP‐seq of BHLHE40 (Figure S5D–E, Supporting Information) indicated that BHLHE40 primarily bound to the promoter and intergenic regions, suggesting that it may play multiple roles in the transcriptional regulation of target genes (Figure 3C). In total, 187 genes were identified as an intersection group between the RNA‐ and BHLHE40 ChIP‐seq datasets. Among these, SREBF1 attracted our interest (Figure 3A and Figure S5F, Supporting Information) because it regulates the key factors involved in fatty acid synthesis and is overexpressed in several cancer types.^[^ ^10^ ^]^ The results of BHLHE40 ChIP‐seq (Figure 3D) and luciferase reporter assays (Figure 3E) demonstrated that BHLHE40 binds to the promoter regions of SREBF1. Furthermore, ChIP‐qPCR of H3K4me3 was performed to confirm the activation of the promoter of SREBF1 (Figure 3F).

Similar to other cancer types, PCa exhibited the upregulation of SREBF1 (Figure 3G).^[^ ^23^, ^24^ ^]^ Higher SREBF1 expression was associated with a reduced overall survival (Figure 3H). As expected, BHLHE40 silencing decreased the expression of SREBF1 and its downstream targets in PCa cells and organoids (Figure 3I–J and Figure S5H, Supporting Information). Key enzymes involved in fatty acid metabolism, such as SCD1, LSS, and HMGCS1, showed increased protein levels in MIA PaCa‐2 cells with BHLHE40 overexpression (Figure S5I, Supporting Information). Functionally, SREBF1 silencing reduced the proliferation of PCa cells (Figure S6A,B, Supporting Information). SREBF1 overexpression in BHLHE40‐silenced cells rescued the viability of both PANC‐1 and BxPC‐3 cells (Figure S6C–F, Supporting Information). Immunofluorescence (IF) assays showed that BHLHE40 co‐localized with SREBF1 in malignant ductal cells derived from PTO1 cells (Figure 3K). Overall, BHLHE40 regulated the transcription of SREBF1, a key factor in fatty acid metabolism in PCa.

### 2.4 Phase‐Separated BHLHE40 Condensates Facilitate the Linking of the Enhancer and Promoter Regions of the SREBF1 Locus

**Figure 4:**
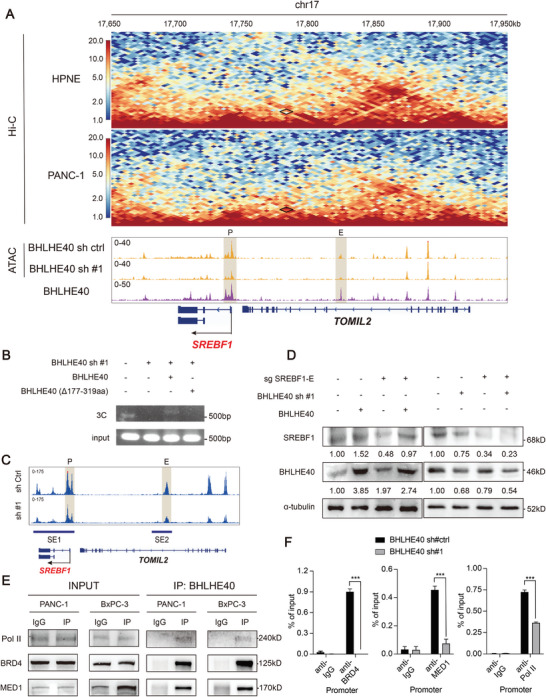
BHLHE40 facilitates the linking of the enhancer and promoter regions upstream of the SREBF1 locus. A) In situ Hi‐C maps of PANC‐1 and HPNE cells, ATAC‐seq of PANC‐1 cells with or without BHLHE40 knockdown, and BHLHE40 ChIP‐seq tracks surrounding the SREBF1 locus. Dots with a black diamond show the potential enhancer‐promoter loops. B) 3C assays were performed to evaluate the existence of an intrachromosomal loop between the promoter and super‐enhancer regions of SREBF1 in PANC‐1 cells when BHLHE40 was knocked down or recovered with wild type or mutant BHLHE40. Nonligated DNA was the negative control. C) Rank ordering of super‐enhancers (ROSE) algorithm was used to predict the putative super enhancers mediated by BHLHE40 via comparing the differential H3K27ac peaks near SREBF1 in BHLHE40 knockdown cells with controls. D) BHLHE40 overexpression could not rescue the SREBF1 expression levels in the sgSREBF1‐E. E) Interactions of BHLHE40 with Pol II, BRD4, and MED1 were verified using immunoprecipitation (IP) assay. F) BRD4, MED1, and Pol II enrichment at the promoter region of SREBF1 in BHLHE40 knockdown or control PANC‐1 cells were determined via ChIP‐qPCR assays. Data presentation: F) data were the mean ± s.d. of *n* = 3 independent experiments. Statistical analysis: unpaired two‐sided t‐test for (F); *P < 0.05, **P < 0.01, ***P < 0.001.

Enhancers or superenhancers flank SREBF1 loci in squamous cancer cells.^[^ ^23^ ^]^ Analysis of Hi‐C sequencing data in PANC‐1 cells revealed an intrachromosomal loop between the promoter and potential enhancer regions of SREBF1; however, this loop was not obvious in an immortalized normal pancreatic epithelial cell line (HPNE) (**Figure** **4**A). Given that BHLHE40 binds upstream of the SREBF1 locus, we investigated whether BHLHE40 contributes to the intrachromosomal loop of SREBF1. BHLHE40 silencing decreased chromatin openness, particularly in the promoter and enhancer regions of SREBF1, of which BHLHE40 was bound (Figure 4A and Figure S7A,B, Supporting Information). Comparing BHLHE40 knockdown cells and controls, the overlapping genes between the group of DEGs in the mRNA‐seq and that of genes with hypo‐accessible regions in ATAC‐seq were enriched in cancer‐related pathways and the regulation of lipid metabolic processes (Figure S7C, Supporting Information). 3C assays were performed to confirm the presence of an intrachromosomal loop between the enhancer and promoter regions of SREBF1 in PANC‐1 cells. Notably, this loop was not detected after BHLHE40 silencing (Figure 4B), suggesting that it was mediated by BHLHE40. Moreover, BHLHE40 silencing decreased H3K27ac modification at the genomic level (Figure S7D, Supporting Information), particularly upstream of the SREBF1 locus, thus further confirming the regulation of BHLHE40 on the transcription of SREBF1 (Figure 4C). As expected, H3K27ac and chromatin accessibility changes in the direct targets of BHLHE40 (LYNX1, DBP, and HDAC5) were also affected by BHLHE40 silencing (Figure S8A–C, Supporting Information). However, the decreased protein level of SREBF1 due to the removal of a 17‐kb segment of the putative enhancer upstream of the SREBF1 locus, which was predicted by the Rank Ordering of Super‐Enhancers (ROSE) algorithm (Figure 4C), could not be completely recovered by BHLHE40 overexpression (Figure 4D and Figure S7E, Supporting Information).

Next, we investigated whether classic coactivators of super‐enhancer formation, such as bromodomain‐containing 4 (BRD4), mediator complex subunit 1 (MED1), and RNA polymerase II (Pol II), contribute to the BHLHE40‐mediated enhancer–promoter loop. Coimmunoprecipitation (Co‐IP) confirmed the interaction of BHLHE40 with BRD4, MED1, and Pol II (Figure 4E). ChIP‐qPCR also showed that these coactivators bind to the promoter and enhancer regions of SREBF1 in a BHLHE40‐dependent manner (Figure 4F and Figure S7F, Supporting Information). BHLHE40 links the promoter and enhancer regions to form an intrachromosomal loop upstream of the SREBF1 locus.

**Figure 5:**
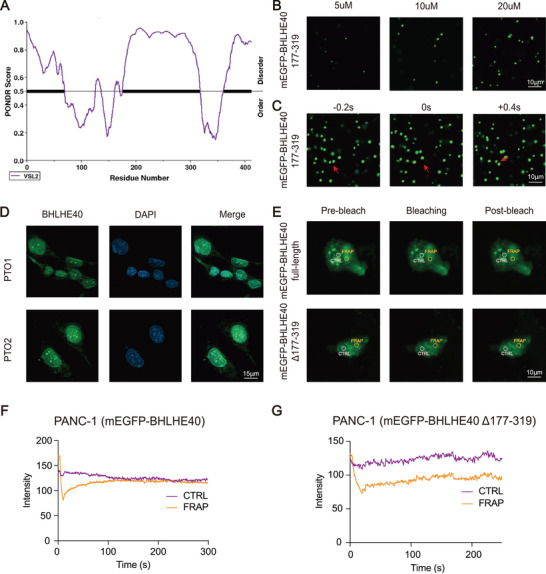
BHLHE40 forms phase‐separated condensates in vitro and in vivo. A) Plotting of intrinsic disorder for BHLHE40 using PONDR VSL2 displays the amino acid location (*x*‐axis) and PONDR VSL2 score (*y*‐axis). Black bar designates the IDR under investigation. B) Representative images of droplet formation at different protein concentrations. BHLHE40 (177–319) was added to the droplet formation buffer to the final concentrations indicated. Scale bar, 10 µm. C) Time‐lapse images of the purified BHLHE40 (177–319) showed that a droplet fusion event occurred. Scale bar, 10 µm. D) Immunostaining of endogenous BHLHE40 (green) and DNA (4,6‐diamidino‐2‐phenylindole (DAPI), blue) in the indicated PTO1 and PTO2. E) Images from the fluorescence recovery after photobleaching (FRAP) experiment with overexpressed mEGFP‐BHLHE40 FL and mEGFP‐BHLHE40 (177–319) cells. Scale bar, 20 µm. F,G) Quantification of FRAP data for mEGFP‐BHLHE40 (full length) and mEGFP‐BHLHE40 (Δ177–319aa) puncta. Bleaching occurs at *t* = 0 s, and background‐subtracted fluorescence intensities are plotted relative to a pre‐bleach time point (*t* = −3 s) for both the bleached area and unbleached control.

The predictor of natural disordered regions (PONDRs) score showed that BHLHE40 contained large IDRs (fragments of 177‐319 amino acids) (**Figure** **5**A). Because IDRs enable the formation of phase‐separated droplets,^[^ ^8^ ^]^ we investigated whether BHLHE40 and its IDRs could form phase‐separated condensates. The recombinant mEGFP, mEGFP‐BHLHE40 (full length, FL), mEGFP‐BHLHE40 (177‐319aa), and mEGFP‐BHLHE40 deleted with 177‐319aa [termed as BHLHE40 (Δ177‐319aa)] proteins were purified and used for the droplet formation assay with varying concentrations (5–20 × 10^−6^ m) (Figure S9A, Supporting Information). mEGFP‐BHLHE40 (177‐319aa) formed spherical droplets in a concentration‐dependent manner (Figure 5B). Droplets formed by mEGFP‐BHLHE40 or mEGFP‐BHLHE40 (177‐319aa) gradually fused to form larger and brighter droplets (Figure 5C). In contrast, BHLHE40 (Δ177‐319aa) did not form droplets (Figure S9B, Supporting Information). Using 2D cultured PTO1 and PTO2 cells, the punctate distribution of BHLHE40 was observed within the cell nucleus (Figure 5D). The dynamic recombination and fast exchange kinetics of the liquid‐like condensates formed by mEGFP‐BHLHE40 were determined by measuring the fluorescence recovery after photobleaching (FRAP). After photobleaching, the mEGFP‐BHLHE40 puncta recovered fluorescence within seconds (Figure 5E–G). The results of the 3C assays showed that full‐length BHLHE40 could facilitate forming the intrachromosomal loop, while BHLHE40 (Δ177‐319aa) could not (Figure 4B and Figure S9C, Supporting Information). These results suggest that phase‐separated BHLHE40 condensates facilitate the linking of the enhancer and promoter regions of the SREBF1 locus, thereby promoting the transcription of SREBF1.

### 2.5 SREBF1 Upregulation Induced by BHLHE40 Protects PCa Cells from Ferroptosis via SCD1

**Figure 6:**
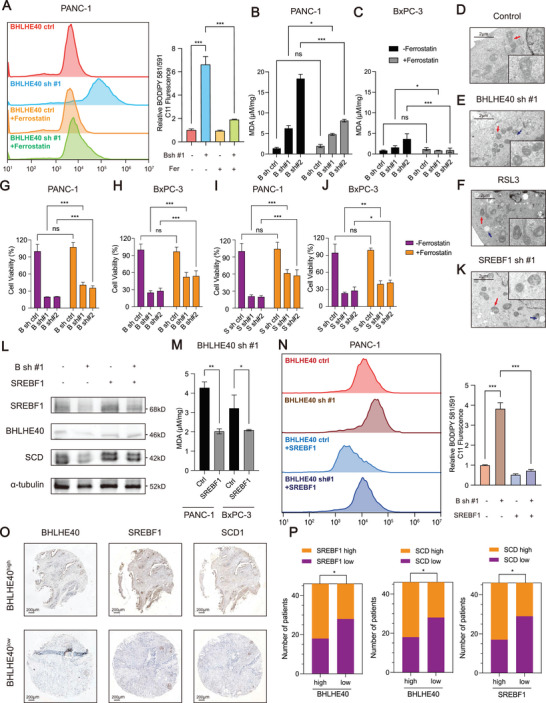
Loss of BHLHE40 promotes ferroptosis in PCa via SREBF1 in vivo. A) BODIPY 581/591 C11 was detected before or after the knockdown of BHLHE40 in the PANC‐1 cell lines in the presence or absence of 2 µmol L^−1^ of ferrostatin‐1. B,C) Malondialdehyde (MDA) was detected before or after the knockdown of BHLHE40 in PANC‐1 and BxPC‐3 cells in the presence or absence of 2 µmol L^−1^ of ferrostatin‐1. D–F,K) TEM imaging was conducted in BHLHE40 or SREBF1 knockdown PANC‐1 cells. RSL3 (2 µmol L^−1^)‐treated PANC‐1 cells functioned as the positive control. Red arrows mark the shrunken mitochondria, and blue arrows mark the lipid droplets. Scale bar, 2 µm. G–J) BHLHE40 or SREBF1 silencing in PANC‐1 or BxPC‐3 cells were treated with ferrostation‐1 (2 µmol L^−1^) for 72 h, and cell viability was evaluated. L) SREBF1 and SCD1 were detected in PANC‐1 or BxPC‐3 BHLHE40 sh #1 cells with the overexpression of SREBF1. M,N) MDA and BODIPY 581/591 C11 were detected in PANC‐1 or BxPC‐3 BHLHE40 sh#1 cells with the overexpression of SREBF1. O) Representative IHC images of BHLHE40, SREBF1, and SCD1 in tissues of patients. P) Correlation between the levels of BHLHE40 and SREBF1, BHLHE40 and SCD1, or SREBF1 and SCD1. Data presentation: B,C,G,H,I,J,M) data were the mean ± s.d. of *n* = 6 independent experiments; A,N) data were the mean ± s.d. of *n* = 3 independent experiments. Statistical analysis: unpaired two‐sided t‐test for A–C, G‐J,M,N); Fisher's exact test for (P); *P < 0.05, **P < 0.01, ***P < 0.001.

Because BHLHE40 and SREBF1 play important roles in apoptosis in other cancer cells,^[^ ^25^ ^]^ we examined whether BHLHE40 or SREBF1 knockdown could increase apoptosis in PCa cells. The knockdown of BHLHE40 or SREBF1 induced apoptosis (Figure S10A, Supporting Information); however, necroptotic or apoptotic inhibitors (Necrostatin1 and Z‐VAD‐FMK, respectively) did not fully recover the cell viability affected by the knockdown (Figure S10B,C, Supporting Information). Because BHLHE40 and SREBF1 contributed to the dysregulation of fatty acid metabolism in PCa (Figure 3B,C), the role of BHLHE40 in peroxidation via SREBF1 was investigated. Flow cytometry using the BODIPY 581/591 C11 probe to detect oxidized lipids revealed that BHLHE40 knockdown increased lipid peroxidation (**Figure** **6**A). The level of MDA was also enhanced in BHLHE40 knockdown cells (Figure 6B,C). In addition, cells with BHLHE40 or SREBF1 knockdown exhibited shrunken mitochondria and increased lipid droplets, similar to iron‐dependent oxidative cell death induced by RAS‐selective‐lethal‐3 (RSL3) (Figure 6D–F). The ferroptosis inhibitor ferrostatin‐1 (fer) at least partially abolished the enhanced lipid peroxidation (Figure 6A–C) and recovered the cell viability decreased by BHLHE40 knockdown (Figure 6G,H). In addition, SREBF1 silencing promoted ferroptosis in PCa cells (Figure 6I–K), and fer treatment reversed the decrease in lipid peroxidation caused by SREBF1 silencing (Figure S10D–F, Supporting Information).

To investigate whether SREBF1 was required for BHLHE40 to inhibit ferroptosis, SREBF1 was overexpressed in PCa cells with BHLHE40 knockdown (Figure 6L). SREBF1 overexpression relieved lipid peroxidation induced by BHLHE40 knockdown (Figure 6M,N) and recovered cell viability (Figure S10G, Supporting Information). Furthermore, the antioxidant NAC was used to recover the malignancy of subcutaneous tumors, confirming the lipid oxidant features after BHLHE40 knockdown (Figure S11A,B, Supporting Information). TEM was performed on xenograft tumors and organoids, which showed that the cells exhibited shrunken mitochondria and increased lipid droplets in vivo (Figure S11C–E, Supporting Information). PTO1 and PTO2 recapitulated the results found in the cell lines (Figure S11F–K, Supporting Information). SCD1 alters lipid membrane composition, reduces lipid peroxidation, and induces ferroptosis^[^ ^26^ ^]^ and is a downstream target of SREBF1; therefore, the expression level of BHLHE40 was associated with those of SREBF1 and SCD1 in PCa clinical samples (Figure 6O,P). Thus, BHLHE40 primarily protects PCa cells from ferroptosis by upregulating SREBF1 expression.

### 2.6 SREBF1 Inhibitor (Fatostatin) Exhibits Remarkable Antitumor Efficacy in PCa with High BHLHE40 Expression

**Figure 7:**
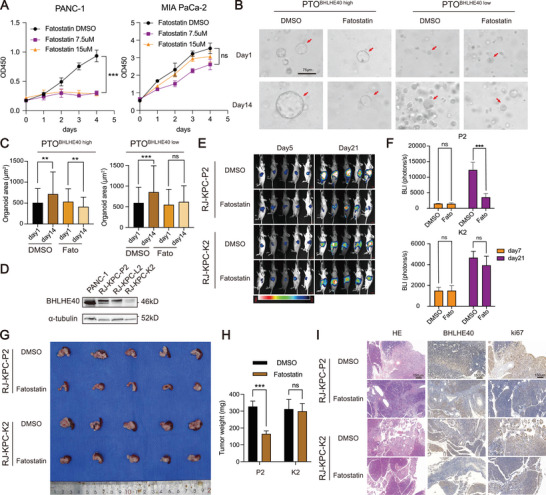
Specific inhibitor of SREBF1 (fatostatin) exerts antitumor efficacy in PCa. A) CCK‐8 analysis of PANC‐1 and MIA PaCa‐2 cells with a gradient concentration of fatostatin. B,C) Representative images of PCa organoids (PTO1 and PTO2, with high or low expression of BHLHE40, respectively) treated with fatostatin or DMSO for 14 d. Diameters of organoids in the two groups were compared and calculated as the mean ± SD. Scale bar, 75 µm. D) BHLHE40 expression in PANC‐1 and KPC mouse‐derived tumor cell lines. E) Representative bioluminescence imaging (BLI) showing tumor growth with or without fatostatin treatment as determined using serial BLI. For each tumor, the BLI values were normalized to the corresponding day zero (immediately before treatment) value. F) Serial photon flux levels in four groups were calculated and compared. G) Indicated tumors established in (E) at necropsy. H) Tumor weight of orthotopic tumors formed by KPC‐K2 or KPC‐P2 receiving treatment with vehicle control or fatostatin for 21 d (*n* = 5 mice per group). I) Representative H&E and IHC staining of BHLHE40 and Ki67 in treated xenografts. Scale bar, 150 µm. Data presentation: A) data were the mean ± s.d. of *n* = 6 independent experiments; C) data were the mean ± s.d. of *n* = 20 independent experiments per time point; F,H) data were the mean ± s.d. of *n* = 5 independent experiments. Statistical analysis: unpaired two‐sided t‐test for (C,F,H); two‐way ANOVA for (A); *P < 0.05, **P < 0.01, ***P < 0.001.

Although the SREBF1 inhibitor fatostatin has been reported to suppress tumor growth and activity,^[^ ^27^ ^]^ its effect on PCa cells has not yet been characterized. The viability of PANC‐1 cells was significantly decreased by fastostatin treatment in a dose‐dependent manner; however, no significant difference was observed in the viability of MIA PaCa‐2 cells after fastostatin treatment, which may be due to the low expression of BHLHE40 in MIA PaCa‐2 cells (**Figure** **7**A). Similar results were observed in patient‐derived organoids (Figure 7B,C) and in vivo pancreatic orthotopic models of KPC mouse cell lines (Figure 7D–H). Additionally, IHC staining showed that orthotopic tumors induced by KPC mouse cells with higher expression levels of BHLHE40 exhibited significantly decreased Ki‐67 expression levels compared to those induced by cells with lower expression levels (Figure 7I). Therefore, these data indicate that the SREBF1 inhibitor has significant antitumor effects on PCa that are dependent on the expression levels of BHLHE40.

## 3 Discussion

PCa is among the deadliest malignancies because of its high rates of recurrence and metastasis. A previous study indicated that lipid metabolism was the most significantly enriched metabolic pathway in PCa with a progressive disease compared to those with a complete response.^[^ ^28^ ^]^ Thus, lipid metabolism may be the Achilles’ heel of PCa treatment. Promising results have been obtained for improving the therapeutic responsiveness by targeting metabolic pathways in various cancer types.^[^ ^29^ ^]^ Lipid metabolism not only promotes cell apoptosis by modulating cell membrane permeability and activating different enzymes,^[^ ^30^ ^]^ but also induces ferroptosis via the iron‐dependent peroxidation of membrane lipids.^[^ ^31^ ^]^ The master regulators of lipogenesis (e.g., SREBF1 and SCD1) increase the cellular levels of saturated lipids and decrease the overall proportion of polyunsaturated fatty acid phospholipids in the plasma membrane, rendering tumor cells resistant to lipid peroxidation and ferroptosis.^[^ ^10^, ^12^ ^]^

The behavior of PCa cells is highly dependent on master regulators, which are acquired in multi‐step tumorigenesis over long‐term exposure to high‐risk factors, and remains critical to the increased viability and proliferation of cells in a fully neoplastic state.^[^ ^1^ ^]^ The activation of SREBF1 has been reported in numerous cancer types and is associated with enhanced cell proliferation, migration, invasion, metastasis, and chemotherapeutic resistance.^[^ ^10^, ^32^ ^]^ SREBF1 enhances the fatty acid desaturation capacity of cancer cells via SCD1‐mediated desaturation. Monounsaturated fatty acids (MUFAs) restore cell growth in SREBF1‐inhibited cells.^[^ ^33^ ^]^ Moreover, exogenous MUFAs inhibit the oxidative death process of ferroptosis by blocking the accumulation of lipid ROS, particularly in the plasma membrane.^[^ ^34^ ^]^

As mentioned previously, insulin signaling via PI3K‐AKT‐mTORC1‐SREBF1 is a critical anabolic pathway for tumor cells to regulate lipogenesis in response to a nutrition‐deprived state.^[^ ^10^ ^]^ Herein, we found that the mTOR signaling pathway also upregulates the expression of BHLHE40, a transcription factor required for the induction of SREBF1 mRNA in rodent liver.^[^ ^21^ ^]^ However, the mechanism underlying the effects of BHLHE40 on SREBF1 expression remains unknown in PCa. In this study, the expression levels of BHLHE40, SREBF1, and SCD1 were found to be correlated (Figure 6O,P). Considering that BHLHE40 is a transcription factor, BHLHE40 silencing reduced SREBF1 expression (Figure 6L). Notably, the IDRs in BHLHE40 linked the enhancer and promoter regions upstream of the SREBF1 locus by forming phase‐separated concentrates (Figure 5B–G and Figure S9B, Supporting Information). An intrachromosomal loop upstream of the SREBF1 locus was identified using Hi‐C in PCa cells and was further confirmed by 3C assays. Thus, we demonstrated that the formation of BHLHE40 phase‐separated condensates contributes to SREBF1 upregulation, leading to dysregulated fatty acid metabolism and subsequent ferroptosis in PCa. Inhibitors targeting lipogenic enzymes regulated by SREBF1, such as FASN and ACSLs, have been investigated in preclinical trials and evaluated for tumor growth, angiogenesis, and metastasis incidence in mouse carcinogenesis models; however, problematic side effects occurred.^[^ ^35^ ^]^ The SREBF1 inhibitors fatostatin and betulin suppressed tumor growth and activity in previous studies.^[^ ^36^ ^]^ Herein, fatostatin showed significant suppressive effects on tumor growth in PCa, with higher expression levels of BHLHE40 (Figure 7E–I). Hence, considering the important roles of lipid metabolism in PCa development and progression, our studies on the BHLHE40‐SREBF1‐SCD1‐ferroptosis axis may provide novel insights into the mechanisms of fatty acid synthesis and desaturation in PCa and facilitate the development of new therapeutic targets for the effective management of PCa.

In summary, we report that chromatin openness‐induced BHLHE40 expression was significantly upregulated in tumors. The oncogenic role of BHLHE40 was characterized using in vitro and in vivo functional assays. Containing large IDRs, BHLHE40 not only regulates SREBF1 transcription as a traditional transcription factor but also links the enhancer and promoter regions of SREBF1 by forming a liquid–liquid phase separation. The detailed mechanism by which BHLHE40 upregulates SREBP provides new insights into the PI3K‐AKT‐mTORC‐SREBP signaling axis. Moreover, the roles of the BHLHE40‐SREBF1‐SCD1‐ferroptosis axis in PCa progression were characterized for the first time, which may shed light on the development of novel therapeutic strategies for PCa that target fatty acid synthesis and desaturation.

## 4 Experimental Section

### Generation of Organoids from PCa Surgical Samples

Following surgical resection, fresh human PCa tissues were processed within 30 min, washed, and cut into small sections using sterile scissors. Collagenase IV was added to a solution with a final concentration of 1.5 mg mL^−1^ and digested in a water bath maintained at 37 °C. The diluted digested solution was filtered through a 40 µm filter and centrifuged at 200 g for 5 min. The pellet was dissolved in serum‐free Dulbecco's modified Eagle medium (DMEM) and mixed with an equal volume of Matrigel. The mixed solution was aspirated, transferred to a microplate, and placed in an incubator for 10–30 min until it solidified. DMEM/F12 containing 2% B27, N‐acetyl‐l‐cysteine (1.25 × 10^−3^ m), epidermal growth factor (EGF) (50 ng mL^−1^), A83‐01 (200 × 10^−9^ m), Noggin (100 ng mL^−1^), R‐spondin 1 (500 ng mL^−1^), Y‐27632 (10 × 10^−3^ m), and dihydrotestosterone (1 × 10^−9^ m) were added to the matrix. On day one, 2000 crypts were placed in each well for organoid development tests, and the quantity and size of the organoids were assessed on day seven.

### Cell Culture and Transfection

The primary PCa cell lines, PANC‐1, BxPC‐3, and MIA PaCa‐2 were purchased from American Type Culture Collection (ATCC) (https://www.atcc.org/). All cell lines were authenticated high‐resolution small tandem repeat profiling and cultured under specified standard conditions. Transient transfections were performed using Lipofectamine 3000 (Invitrogen, USA) following the instructions from ATCC.

### Cell Migration Detection

Transwell (Costar, USA) assays were performed to gauge cell migration capacity. Cells (3 × 10^4^) were layered in the upper chamber with serum‐free culture medium, while 10% fetal bovine serum culture medium was placed in the lower chamber. After removing non‐migrating cells from the top chambers after 48 h, the cells underneath the filter were stained with crystal violet and counted under a microscope at three separate sites.

### RNA Extraction and mRNA‐Seq Library Preparation

Total RNA was extracted from the harvested cells using TRIzol Reagent (Thermo Fisher Scientific, USA). One microgram of RNA per sample was used for RNA quality control (Qubit 3.0 Fluorometer, Thermo Fisher Scientific, USA) and for subsequent steps. Sequencing libraries were generated according to the manufacturer's instructions using the NEB Next Ultra Directional RNA Library Prep Kit (Illumina, NEB, USA). The total RNA integrity was assessed (Agilent 2100 Bioanalyzer of Agilent Technologies, USA) using the RNA Integrity Number. Library fragments were purified using the AMPure XP system (Beckman, USA) to select cDNA fragments of 200–300 bp. Finally, the Illumina HiSeq X Ten platform with paired‐end 150 bp reads was used to sequence the libraries.

### Spearman Correlation Analysis of Rapamycin IC_50_ Scores and BHLHE40 Gene Expressions

PAAD was downloaded from the TCGA dataset (https://portal.gdc.com) for expression profiles and clinical data. Using the pharmacogenomics database, Genomics of Drug Sensitivity in Cancer (GDSC), and R package “pRRophetic,” the response for rapamycin was forecasted. Ridge regression was used to estimate the half‐maximal inhibitory concentration (IC_50_) of each sample using default values. All the above methods and R package were implemented using the R Foundation for Statistical Computing (2020) version 4.0.3.

### Analysis of Data from TCGA and the Genotype‐Tissue Expression (GTEx) using GEPIA

The correlation between BHLHE40 and EP300, survival curve according to BHLHE40 expression, and mRNA expression of transcription factors from PCa tumor samples in TCGA and normal pancreatic tissue in GTEx were analyzed using GEPIA^[^ ^37^ ^]^ (http://gepia2.cancer‐pku.cn).

### Chromatin Immunoprecipitation (ChIP) Library Generation and Sequencing

The ChIP assay was performed as previously described.^[^ ^38^ ^]^ In this study, 20 × 10^6^ adhered cells were lysed with RIPA buffer to prepare nuclear extracts. After chromatin shearing using sonication, the nuclear lysates were incubated with Protein A Dynabeads (Invitrogen, USA) at 4 °C overnight coupled with 3–5 µg of antibody for preparing each sample. The following antibodies were commercially acquired: anti‐histone H3 (acetyl K27) (ab4729, 2 µg/25 µg of chromatin, Abcam), anti‐BHLHE40 (NB‐1800, 5 µg/25 µg of chromatin, Novus), and anti‐Histone H3 (tri methyl K4) antibodies (ab8580, 5 µg/25 µg of chromatin, Abcam). Following immunoprecipitation, the beads were collected and washed. DNA was eluted, de‐crosslinked, and purified using the QIAquick PCR Purification Kit (Qiagen, USA) under protocol of the manufacturer. For each sample, 5–10 ng of purified ChIP DNA was used as the input material for the sequencing library using a VAHTS Universal DNA Library Prep Kit for Illumina V3 (Vazyme Biotech, China) and then sequenced using 150 bp paired‐end reads on an Illumina HiSeq X Ten platform.

### CUT&Tag Library Generation and Sequencing

According to the manufacturer's protocol, CUT&Tag was performed as previously described^[^ ^39^ ^]^ using the Hyperactive Universal CUT&Tag Assay Kit for Illumina (Vazyme Biotech, TD903). Briefly, the cells were incubated with 10 µL of prewashed ConA and 0.5 µg of antibody for 2 h at room temperature. The beads were washed twice with 50 µL of Dig‐Wash buffer and then co‐cultured with 0.5 µg of a secondary antibody for 30 min. After washing, 300 µL of tagmentation buffer was added, and the sample was incubated at 37 °C for 1 h. The reaction was stopped using 10 µL of 0.5 m EDTA, 2.5 µL of 20 mg mL^−1^ Proteinase K, and 3 µL of 10% SDS and then purified using the QIAquick PCR Purification Kit (QIAGEN, USA). After amplification, all libraries were sequenced using Illumina HiSeq X Ten with a 150 bp paired‐end reading strategy.

### Nuclei Isolation and ATAC‐Seq Library Preparation

ATAC‐seq libraries were prepared as described previously^[^ ^40^ ^]^ with certain modifications. Briefly, 1 × 10^4^ cells were lysed in 100 µL of cold lysis buffer [10 × 10^−3^ m Tris‐HCl (pH 7.4), 3 × 10^−3^ m MgCl_2_, 10 × 10^−3^ m NaCl, and 0.1% IGEPAL CA‐630 (Sigma‐Aldrich, USA)] on ice for 15 min to isolate the nuclei, followed by centrifugation at 500 × *g* for 10 min at 4 °C. After removing the supernatant, the cell nuclei pellet was resuspended in 50 µL of Tn5 transposition reaction mix (Vazyme Biotech, China) and incubated for 30 min at 37 °C. The samples were then purified using the QIAquick PCR Purification Kit (QIAGEN, Germany). PCR was used for library amplification under the following conditions: 72 °C for 3 min; 98 °C for 30 s; thermocycling at 98 °C for 15 s, 60 °C for 30 s, and 72 °C for 1 min; and 72 °C for 5 min. After using VAHTS DNA Clean Beads (Vazyme Biotech, China) to purify the PCR product, the ATAC‐seq libraries were sequenced on an Illumina HiSeq X Ten system with 150 bp paired‐end reads for each sample.

### Data Processing for mRNA Sequencing

As previously described,^[^ ^40^ ^]^ FastQC (v0.11.6) was used for the quality control assessment of the output. The sequences were aligned with the *Homo sapiens* hg19 reference sequence using STAR aligners.^[^ ^41^ ^]^ The SAMtools view‐s option was used for all the samples to generate BAM files that contained a similar number of sequencing reads. The mapped reads were converted to fragments per kilobase of exons per million fragments mapped using Cuffdiff 2.1.1^[^ ^42^ ^]^ to generate the gene expression profiles. Differentially expressed genes (DEGs) were filtered to obtain a *P* value < 0.05 and the absolute value of log2 fold change (FC) > 2 in at least one of the two groups.

### Data Processing for ChIP Sequencing and CUT&Tag Data

Quality control of each sample was performed using FastQC (v0.11.6). All ChIP‐seq and CUT&Tag datasets were trimmed and aligned to human genome build 19 (hg19) using Bowtie2.^[^ ^43^ ^]^ After alignment, duplicate reads were removed using the SAM tools. MACS2 was used to identify the ChIP‐seq and CUT&Tag enrichment peaks in the background.^[^ ^44^ ^]^ For the histone modification of H3K27ac, the parameters were modified to facilitate the accurate detection of broad peaks using the following parameters: broad‐broad‐cutoff 1E‐3 ‐p 1E‐3. DeepTools 2.0 was used to generate Bigwig files, heatmaps, and average profiles for normalization and visualization of the sequencing data.

### Data Processing for ATAC‐seq

Raw ATAC‐seq data were assessed for quality control (FastQC v0.11.6), aligned to hg19 (Bowtie2), and processed to remove duplicate reads (SAMtools)^[^ ^45^ ^]^ for the subsequent generation of BAM files. BAM files were also converted to the BigWig format using the “bamCoverage” scripts in DeepTools 2.0. To determine the distribution of fragment sizes, paired‐end sequencing was performed using Picard Tools (v2.9.4). For peak identification of the chromatin accessible regions, the module “callpeak” in MACS2 (v2.1.2) was used with the parameters—extsize 200‐shift−100. The “annotatePeaks.pl” script in HOMER (v4.10)^[^ ^46^ ^]^ was applied to match the location of a given peak with its genomic features [Promoter‐TSS (from −1000 to +1000 bp of transcription start site), TTS (from −100 to + 1000 bp of transcription termination site), 5′ UTR, 3′ UTR, Exon, Intronic, or Intergenic regions]. Heatmap figures and average profiles were generated using “computeMatrix,” “plotHeatmap,” and “plotProfile” functions.

### Hi‐C Analysis and Chromosome Conformation Capture Assay (3C) Experiment

The raw sequenced Hi‐C reads from the public dataset GSE149103 were first mapped to hg19 using the BWA‐MEM algorithm,^[^ ^47^ ^]^ of which invalid Hi‐C reads were discarded, and then processed using the HiCExplorer suite of tools (v3.6).^[^ ^48^ ^]^ Quality control measures for all samples were performed using the HiCExplorer application hicQC. The hicBuildMatrix function was then used to generate raw Hi‐C matrices at a 5 kb resolution. The Hi‐C matrices of replicates were merged, normalized, and corrected^[^ ^49^ ^]^ using hicSumMatrices, hicNormalize, and hicCorrectMatrix. Heatmaps were generated to visualize the interaction frequencies at different resolutions using hicPlotMatrix. Next, a 3C assay was performed to confirm the existence of the enhancer–promoter loop upstream of the SREBF1 loci, as previously reported.^[^ ^50^ ^]^ Cross‐linked nuclear DNA of 2 × 10^6^ PANC‐1 cells with or without relevant gene manipulation was extracted, digested with the restriction enzyme BspHI, and ligated with T4 ligase. The 3C products were amplified using PCR with a pair of primers shown in Table S3 (Supporting Information).

### Identification of Differentially Accessible Regions (DARs)

To evaluate changes in chromatin accessibility, DeepTools was used to compute the average RPKM normalized values for the ATAC‐seq peaks. Hyper‐accessible regions (hyper) corresponded to increased ATAC‐seq signals, with an average fold change >1.5 compared to that for the control sample. In contrast, the hypo‐accessible regions (hypo) were associated with decreased ATAC‐seq signals, which showed an average fold change >1.5 in controlled samples compared to experimental samples. The heatmaps and average profiles of regions that were expanded to ±1.5 kb surrounding the DARs center were generated using the “plotHeatmap” and “plotProfile” functions in DeepTools.

### Pathway Enrichment Analysis

For the functional enrichment of gene sets, the enrichment analysis and visualization of Gene Ontology (GO) terms and Kyoto Encyclopedia of Genes and Genomes (KEGG) pathways were performed using the R package “clusterProfiler.”^[^ ^51^ ^]^ Pathways with *P* value <0.05 were considered significantly enriched.

### Gene set Enrichment Analysis (GSEA)

The log2 ranked genes (fold change) and GSEA (v4.1.0)^[^ ^50^ ^]^ were performed using ranked lists with Hallmark, GO, and KEGG gene sets downloaded from MsigDB (v7.4, http://software.broadinstitute.org/gsea/msigdb).

### Tissue Specimens, Immunohistochemical (IHC) Staining, and Analysis of Tissue Microarray (TMA)

The clinical tissue samples used in this study were histopathologically and clinically diagnosed at Ruijin Hospital with patient consent and with approval from the Ethics Committee. This information is presented in Table S2 (Supporting Information). IHC staining was performed with the following antibodies: anti‐BHLHE40 (1:100, Novus), anti‐SREBF1 (1:100, Abcam), anti‐SCD1 (1:100, Abcam), and anti‐Ki67 (1:100, Servicebio) to detect protein expression. The protein expressions of BHLHE40, SREBF1, and SCD were analyzed using the digital pathological image analysis software based on artificial intelligence learning (Servicebio Technology) via tracking, color selection, calculation, and TMA. The analysis formula is H‐SCORE = ∑(pi×i) = (percentage of weak intensity ×1) + (percentage of moderate intensity ×2) + (percentage of strong intensity ×3).

### Immunoprecipitation and Western Blotting

To perform the immunoprecipitation experiment, cells were lysed in a 0.5% NP40 solution containing protease and phosphatase inhibitors. The supernatant of centrifuged lysates was treated with antibody‐conjugated agarose for 8 h at 4 °C (12000 rpm for 15 min at 4 °C). Immunoprecipitants were boiled and subjected to western blot analysis following standard procedures.

For western blotting, total proteins were extracted using a lysis buffer containing protease inhibitors and separated using 4%–20% SDS‐PAGE gels. Proteins were then transferred to polyvinylidene difluoride membranes. After blocking with 5% bovine serum albumin (BSA), the membranes were incubated overnight with primary antibodies. The membranes were coated with the secondary antibody for 1 h at room temperature after washing three times in TBS containing 0.1% Tween‐20. The bands were visualized using electrochemiluminescence. The following primary antibodies were commercially acquired: anti‐BHLHE40 (NB‐1800, 1:1000, Novus), anti‐SREBF1 (ab3259, 1:1000, Abcam), anti‐EP300 (#54062, 1:1000, Cell Signaling Technology), anti‐acetyllysine (PTM‐105RM, 1:1000, PTMBIO), anti‐mTOR (A2445, 1:1000, ABclonal), anti‐phospho‐mTOR‐S2448 (AP0094, 1:1000, ABclonal), anti‐histone H3 (acetyl K27) (ab4729, 1:2000, Abcam), anti‐SCD1 (ab19862, 1:1000, Abcam), anti‐FASN (ab128856, 1:1000, ABclonal), anti‐LSS (ab124785, 1:1000, Abcam), anti‐ORP1 (ab131165, 1:1000, Abcam), anti‐HMGCS1 (#36877, 1:1000, Cell Signaling Technology), anti‐RNA polymerase II CTD repeat YSPTSPS (phospho S5) (ab5131, Abcam, 1:1000), anti‐MED1 (A1724, 1:1000, ABclonal), anti‐Brd4 (ab128874, 1:1000, Abcam), anti‐histone H3 (A2348, 1:2000, ABclonal), anti‐alpha tubulin (11224‐1‐AP, 1:2500, Proteintech), and anti‐GAPDH antibodies (60004‐1‐Ig, 1:2500, Proteintech).

### RNA Isolation and Quantitative Real‐Time PCR

Total RNA was extracted using the TRIzol reagent, and the corresponding cDNA was obtained using reverse transcription with the AG Evo M‐MLV RT Mix Kit. Quantitative real‐time PCR was performed using an ABI 7500 real‐time PCR system (Applied Biosystems, USA). The primers used are listed in Table S3 (Supporting Information).

### Colony Formation Assays

The relevant PCa cell lines were cultured at a density of 1500 cells/well for 7 d to perform colony formation tests in six‐well plates. Cell colonies were rinsed in phosphate‐buffered saline (PBS), fixed in pure methanol for 5 min, and stained for 30 min at room temperature using a crystal violet aqueous solution (C0121, Beyotime, China). Before inverting the culture plates to dry, the cells were rinsed three times with PBS. Colonies with >50 cells were counted using the ImageJ software.

### Cell Proliferation and Viability Assays

PCa cell lines transfected with shRNA or control plasmids were seeded in 96‐well plates. Viable cells were evaluated every 24 h for 5 d using Cell Counting Kit 8 (CCK‐8; Dojindo, Japan) according to the manufacturer's protocol. Cell viability was determined using the same reagent after 48 h of incubation, and the results were detected using a Synergy LX multimode reader (Agilent, USA).

### Protein Purification and In Vitro Phase Separation Observation

BHLHE40 and its mutants were labeled with mEGFP. The constructs were transformed into *Escherichia coli* BL21 (DE3) cells. To produce MBP‐fusion proteins, bacteria were cultured for 4 h at 37 °C with 1 × 10^−3^ m of isopropyl β‐D‐1‐thiogalactopyranoside. The OD600 value was set at 0.7. The bacterial cells were resuspended in BC500 buffer, which contained 1 mM DTT, 0.1% NP40, a protease inhibitor cocktail, 500 × 10^−3^ m KCl, 20% glycerol, and 0.2 × 10^−3^ m EDTA. Following centrifugation, Ni‐affinity chromatography was performed to purify the supernatant. The SDS‐PAGE analysis of purified proteins was followed by Coomassie blue staining. The in vitro liquid‐liquid phase separation (LLPS) observations were based on microscopy. A self‐made flow chamber comprising a glass slide, coverslip, and layer of double‐sided tape acting as a spacer was filled with the protein mixture. Images were captured using a 60× oil lens on a Zeiss LSM900 confocal microscope.

### Fluorescence Recovery after Photobleaching (FRAP) Assay

A Zeiss LSM900 confocal microscope was used for the FRAP tests. Live‐cell FRAP experiments were conducted at 37 °C with 5% CO_2_, and the fluorescence signal was bleached for 10 s using 30% maximum power of a 488 nm laser. The bleaching region had a diameter of 1–2 µm, and the recovery was captured at a rate of 2 s per frame. For image acquisition, the intensities were adjusted for global photobleaching (from a nearby unbleached droplet) after subtracting background signals. Finally, the recovery time of each bleached condensate was calculated by fitting the FRAP recovery curves.

### Organoid Cell Vitality Measurement Assays

Cell vitality was measured using the CellTiter‐Glo 3D cell viability assay (Vazyme, China) after patient‐derived organoids were treated with 0.1% DMSO as a control or to fatostatin at doses of 15 × 10^−6^ m for 5 d. As directed by the manufacturer, cells were lysed and incubated at 37 °C for 30 min. Luminescence was measured using a microplate reader (BioTek Synergy, Agilent, USA).

### C11‐BIDOPY Staining

Following the experiments described in previous sections, cells were seeded into six‐well cell culture plates and pretreated with various substances or plasmids. The cells were dissociated, resuspended, cleaned, and stained with 2 mol L^−1^ of C11‐BODIPY for 15 min before undergoing flow cytometry, which was used to measure fluorescence intensity (Beckmann, USA). The cells were seeded onto circular coverslips for confocal imaging and exposed to 2 mol L^−1^ of C11‐BODIPY for 15 min before detection. After washing the cells and fixing them with 4% paraformaldehyde (Sigma), an inverted microscope was used to capture images.

### Dual‐Luciferase Reporter Gene Assays

A dual‐luciferase reporter assay was performed using the Dual‐Luciferase Reporter Assay System E1960 (Promega, USA). Wild‐type or truncated SREBF1 promoters were cloned into the pGL4.10 basic plasmid (Promega, USA). PANC‐1 cells were cotransfected with Firefly and Renilla luciferase‐expressing plasmids and the Lipo3000 reagent (Invitrogen, USA) at a ratio of 0.1 µg:0.02 µg:1 µL. Forty‐eight hours after transfection, cells were lysed with 50 µL of Passive Lysis Buffer (Promega, USA), and luciferase activities were measured from 20 µL of cell lysates using the Dual Luciferase Reporter Assay on a Synergy LX Luminometer (Agilent, USA). Luciferase activity was normalized to that of Renilla luciferase.

### Malondialdehyde (MDA) Detection

Cells were seeded into six‐well cell culture plates before dissociation. Protein concentrations were determined using a BCA protein assay kit (Yeasen, China) following cell homogenization, and MDA was subsequently determined using a lipid peroxidation MDA test kit (ABclonal, China). The ratio of MDA‐to‐protein concentration was determined by calculating the MDA content.

### Transmission Electron Microscopy (TEM) Imaging

PANC‐1 cells were seeded into 10 cm cell dishes for TEM imaging and subsequently treated with the appropriate substances for 24 h. The treated cells were collected and fixed with 2.5% glutaraldehyde. PBS (0.1 m) was used to wash the cells thrice for 15 min each (pH 7.4). After post‐fixing with 1% OsO_4_ in 0.1 m PBS (pH 7.4) for 2 h at room temperature, the cells became photosensitive. The tissues were washed three times for 15 min each in 0.1 m PBS after removing OsO_4_ (pH 7.4). The cells were dehydrated at room temperature, embedded overnight in resin, and allowed to penetrate. A 65 °C oven was used to polymerize the embedding models with resin and samples for >48 h. The resin blocks were removed from the embedded models and stored at room temperature for further use. A 2% uranium acetate‐saturated alcohol solution was incubated for 8 min to prevent light staining, followed by rinsing three times with 70% ethanol and three times with ultrapure water. After avoiding CO_2_ staining for 8 min, 2.6% lead citrate was rinsed three times with ultrapure water. The cuprum grids were then set on a grid board to air dry overnight at room temperature. The cuprum grids were examined using TEM, and images were captured.

### Animal Research (Subcutaneous and Orthotopic Models)

All experiments performed on mice were reviewed and approved by the Review Board of the Ruijin Hospital Ethics Committee for the Use of Living Animals. For the subcutaneous xenograft model, 100 µL cell suspensions (2 × 10^6^ cells) were injected subcutaneously into the upper right flank of BALB/c nude mice in a sterile environment. Subcutaneous tumor nodes were excised after reaching 800–1500 mm^3^.

For the orthotopic model, cell suspensions of the specified cells into the pancreas of four‐week‐old male BALB/c nude mice to produce orthotopic tumors were injected. Intravital imaging was performed using transduction with CMV‐Luc‐PGK‐puro lentivirus (Genechem, China). This enabled the monitoring of tumor development and regression. The normalized photon flux was used to calculate tumor volume. Mice were randomly assigned to the relevant treatment groups when the tumor volume reached 10^7^ photons s^−1^ based on a luminescence signal. The mean tumor volumes in each group were comparable before the commencement of therapy. Following the start of therapy, no mice were excluded. Tumor‐bearing mice were treated with either vehicle (10% DMSO, 40% PEG300, 5% Tween‐80, and 45% saline) or fatostatin (20 mg kg^−1^ ) dissolved in the same reagent twice weekly for three weeks, to test the effects of the drug.

In vivo imaging was used to track tumor development after luciferase‐labeled cells were orthotopically injected into BALB/c nude mice at specific intervals. Mice were anesthetized with 2% isoflurane before being placed in an IVIS‐50 chamber (Tanon, China) in the supine position, and isoflurane was delivered through the nose‐cone ports within the chamber for the duration of the imaging procedure. Throughout the process, the chamber temperature was maintained at 37 °C. Green fluorescence and background fluorescence filters were used to gather light, and Living Image software was used to analyze the photos (Tanon, China). The ratio of the fluorescence photon flux in the region of interest to the fluorescence signal in the background region devoid of cells or tumors has been reported as a specific signal (normalized photon flux).

### 18F‐FDG PET Imaging, Image Reconstruction, and Quantitative Evaluation for the Metastatic Model of PCa

To demonstrate the role of BHLHE40 in PCa metastasis, 2 × 10^6^ PANC‐1 cells with or without BHLHE40 knockdown were injected into the splenic vein of each BALB/c nude mouse. After 4 weeks, the mice were subjected to PET/CT analysis, which was performed on an Inveon Multi‐Modality System (Siemens, Germany). The animals were anesthetized using 2% isoflurane in oxygen gas for administering 0.1 mL [18F]‐FDG with an activity of 10 MBq and anesthetized again 2 h after the injection. Before imaging, the mice were placed on a PET scanning bed near the center of the image and given 1.5% isoflurane in oxygen at 2 L min^−1^. An Inveon Acquisition Workplace 1.5 was used for scanning. Prior to the PET scan, a 10‐min CT X‐ray for attenuation correction was performed with 80 Kv, 500 uA, and 1100 ms of exposure duration. Following the acquisition of 10 min static PET scans, images were reconstructed using a 3D ordered subsets expectation maximum algorithm, followed by maximization/maximum a posteriori provided by IAW. Individual quantification of the [18F]‐FDG uptake in each of the 3D regions of interest (ROIs) was calculated using the Inveon Research Workplace (v4.2) under the guidance of CT images. Mean standardized uptake values (SUVs) were calculated by dividing the relevant ROI concentration by the ratio of liver metastatic lesion activity to body weight. The presence of metastatic lesions was confirmed by a physician majoring in radiology.

### Detection for Apoptosis

After treatment, cells were collected and resuspended in 1% BSA. The cells were then stained with markers using an Annexin V‐FITC/PI Apoptosis Detection Kit (A211‐01, Vazyme, China). The cells were examined using flow cytometry (CytoFLEX S, Beckman, USA) after incubation at room temperature in the dark for 15 min, and analyzed using FlowJo (v10.8.1).

### KPC Mice‐Derived Cell Line Construction

To obtain a primary cell line from tumor‐bearing LSL‐Kras^G12D/+^, LSL‐Trp53^R172H/+^, and Pdx1‐Cre (KPC) mice (Cyagen Biosciences Inc., China), orthotopic tumor tissues were dissected carefully to avoid contamination with the adjacent normal pancreas or other tissues. Dissected tissues were minced and incubated with 0.125 mg mL^−1^ Collagenase Type XI and 0.125 mg mL^−1^ Dispase II for 2 h at 37 °C with moderate rocking. For single cells, tissues were further digested with TrypLE for 10 min at 37 °C with moderate agitation. The cells were then resuspended in DMEM containing 10% FBS, penicillin, and streptomycin, and plated on cell culture plates. These cells were named RJ‐KPC‐P2, RJ‐KPC‐L2, and RJ‐KPC‐K2.

### Statistical Analysis

Statistical analyses were performed using GraphPad Prism version 8.0. Patients in the tissue microarray were divided into low and high BHLHE40, SREBF1 or SCD1 groups according to the 50% cut‐off H‐score value. K–M curve analysis was performed to assess the association with overall survival using SPSS v23 (IBM Inc.). All statistical tests were indicated in the figure legends.

### Ethics Approval and Consent of Participation

The studies were performed in accordance with the Declaration of Helsinki and the International Conference on Harmonisation Guideline for Good Clinical Practice. The Ruijin Hospital Ethics Committee approved the use of the patient samples (No. 2021‐161) and animal study (No. RJ2023047) in this study. All patients have signed written informed consent.

## Conflict of Interest

The authors declare no conflict of interest.

## Author Contributions

Y.C., X.W., Y.L., and P.L. contributed equally to this work. This study was performed by B.S., L.J., Y.C., and X.W. Y.L. and P.L. developed the methodology. Y.L., P.L., Y.Z., S.Z., Y.J., H.C., and D.C. performed the experiments and acquired the data (including providing animals, acquiring and managing patients, and providing facilities). Data analysis and processing (e.g., statistical analysis, bioinformatics, and biostatistics) were conducted by Y.C., X.W., Y.L., L.H., J.L., R.Z., C.P., and X.D. Administrative, technical, or material support (e.g., reporting or organizing data and constructing databases) was provided by Y.C., M.S., L.W., X.T., M.X., J.L., and B.S. The writing, review, and/or revision of the manuscript were performed by Y.C., D.C., L.J., and B.S. Y.C., X.W., D.C., L.J., and B.S. supervised the study. B.S. was the revision director. All the authors have read and approved the final version of the manuscript.

## Supporting information

Supporting Information

Supplemental Table 1

Supplemental Table 2

Supplemental Table 3

Supplemental Figure

## Data Availability

The data that support the findings of this study are available on request from the corresponding author. The data are not publicly available due to privacy or ethical restrictions.
