# Supplementary material for: BHLHE40 Inhibits Ferroptosis in Pancreatic Cancer Cells via Upregulating SREBF1
Source: Adv Sci (Weinh). 2023 Dec 8;11(7):2306298. doi: 10.1002/advs.202306298 (PMC10870036; doi:10.1002/advs.202306298)
Supplement: Supplementary file 1 — Supporting Information [file ADVS-11-2306298-s002.pdf]

ADVANCED SCIENCE
Open Access

Supporting Information

for *Adv. Sci.*, DOI 10.1002/advs.202306298

BHLHE40 Inhibits Ferroptosis in Pancreatic Cancer Cells via Upregulating SREBF1

Yizhi Cao, Xuelong Wang, Yang Liu, Pengyi Liu, Jiejie Qin, Youwei Zhu, Shuyu Zhai, Yongsheng Jiang, Yihao Liu, Lijie Han, Jiaxin Luo, Ronghao Zhang, Minmin Shi, Liwen Wang, Xiaomei Tang, Meilin Xue, Jia Liu, Weishen Wang, Chenlei Wen, Xiaxing Deng, Chenghong Peng, Hao Chen, Dongfeng Cheng, Lingxi Jiang* and Baiyong Shen**

Supplementary files

Supplementary Table S1: Gene list for multi-omics analysis.

Supplementary Table S2: Clinical features of 92 patients from the RJ-cohort.

Supplementary Table S3: Primers for qPCR assays and KPC mouse identification.

Supplementary Figure legends

Figure S1. Histopathological and genomic validations and epi-genomic distributions for pancreatic tumoral and para-tumoral organoids. (A) Hematoxylin and eosin staining showed the internal structures of patient-derived tumor organoids (PTOs) and para-tumor organoids (PNOs). Scale bar, 100 μm. (B) Whole-exome sequencing profiles demonstrated distinct gene mutations in three tumor- and two para-tumor-derived organoids. (C)–(D) CUT&Tag signals show the profiles of H3K27ac peaks in PTOs or PNOs. Signals within 2 kb surrounding the center of gain or loss regions are displayed in descending order. (E) Normalized ATAC-seq signals in PNOs and PTOs over differentially accessible regions (DARs) are represented in the heatmap.

Figure S2. mTOR-induced BHLHE40 expression is correlated with unfavorable prognosis. (A) Examination of TCGA-PAAD and GTEx data revealed a robust connection between BHLHE40 and EP300 mRNA levels (p <0.001). (B)–(C) When PCa cells were treated with C646, a histone acetyltransferase inhibitor that targets EP300, the BHLHE40 mRNA levels decreased in a time- (with a concentration of 10 μM) or dose-dependent (with a 12-h treatment) manner. (D–I) IC_{50} testing of rapamycin across multiple cell lines of PCa. (J) Quantification of EP300 activity in PANC-1 cells treated with or without rapamycin. EP300 was extracted using immunoprecipitation with the anti-EP300 antibody. P53 peptide was used as the substrate with acetyl-CoA. (K) Compared to most other tumor types, the PCa samples had higher levels of BHLHE40 expression, according to a pan-cancer analysis based on 31 tumors. (L) Expressed transcriptional factors ranked by mean log2 fold-change in primary PCa versus adjacent pancreas in GSE16515 datasets. BHLHE40 was labeled and ranked in the front. (M) Elevated mRNA level of BHLHE40 was associated with lower OS in TCGA (n = 179, P = 0.0039). (N) Western blot analysis of

EP300, mTOR, p-mTOR, and BHLHE40 abundances across PCa cell lines. Data presentation: (B), (C) and (J) data were the mean ± s.d. of n = 3 independent experiments; (A), (K) and (M) data was acquired from gepia2.cancer-pku.cn. Statistical analysis: spearman rank correlation test for (A); unpaired two-sided t-test for (B), (C), (J) and (K); log-rank test for (M). *$P < 0.05$, **$P < 0.01$, ***$P < 0.001$.

Figure S3. BHLHE40 is critical for growth and liver metastasis of PCa *in vitro* and *in vivo*. (A) Western blot of BHLHE40 knockdown efficiency in the BxPC-3 cell lines. (B)–(D) Effects of BHLHE40 knockdown on the proliferation ability of BxPC-3 and PANC-1 cell lines were measured using CCK-8 and colony formation assays. Scale bar, 1 cm. (E)–(G) Invasion assay was conducted in shBHLHE40 versus sh ctrl groups in BxPC-3 and PANC-1 cell lines using 24-well Transwell chambers. Cell invasion was assessed by counting the invasion area after 48 h. Scale bar, 200 μm. (H)–(J) Effects of BHLHE40 knockdown on the migration ability of the BxPC-3 cell lines was measured using the wound-healing assay. Scale bar, 100 μm. (K) PCa cells (BxPC-3) stably transfected with BHLHE40 sh ctrl or sh #1 were injected subcutaneously into four-week-old BALB/c male nude mice (n = 5 for each group). Representative images are of dissected xenogeneic tumors from nude mice in each group. (L)–(N) Tumor weights and volumes of PANC-1 and BxPC-3 derived xenografts were measured and calculated after subcutaneous injection. 'B sh' or 'BHLHE40 sh', short for BHLHE40 short hairpin RNA. Data presentation: (B) data were the mean ± s.d. of n = 6 independent experiments; (L), (M) and (N) data were the mean ± s.d. of n = 5 independent experiments; (C), (D), (F), (G) and (J) data were the mean ± s.d. of n = 3 independent experiments. Statistical analysis: two-way ANOVA for (B) and (N); unpaired two-sided t-test for (C), (D), (F), (G), (J), (L) and (M). * $P < 0.05$, ** $P < 0.01$, *** $P < 0.001$.

Figure S4. PET-CT, gross specimen, and H&E staining images of liver metastatic lesions with or without BHLHE40 knockdown. (A–B) Upper row (PET-CT and SUV): PET-CT images for liver metastatic lesions and their mean SUV. Red circle marks liver metastatic lesion. Middle row (gross specimen): Yellow circle marks metastatic lesion at necropsy. Scale bar, 5 mm. Bottom row (H&E staining): Histopathological demonstrations of liver metastatic lesion. Scale bar, 1 mm.

Figure S5. Integrated analysis of ChIP- and mRNA-seq identified SREBF1 as the downstream target of BHLHE40. (A) Differential analysis was conducted in BHLHE40 knockdown or control PANC-1 cells, and the differentially expressed genes (DEGs) were represented in the volcano plot. (B) Bubble plot showing gene ontology (GO) enrichment analysis of downregulated genes related to tumor proliferation and metastasis after BHLHE40 knockdown. (C) Gene set enrichment analyses (GSEA) of fatty acid metabolism and cholesterol comparing the BHLHE40 knockdown group with the control group. (D) Known motifs for BHLHE40 ChIP-seq recognized using HOMER. (E) ChIP-seq summary plot of BHLHE40-binding intensities across BHLHE40 peaks centered on transcriptional starting site (TSS) in the PANC-1 cell lines. (F) Representative images of SREBF1 IHC staining of subcutaneous tumor induced by BHLHE40 knockdown or control cells. (G) H-score evaluation and statistics of SREBF1 IHC staining images. (H) RNA levels of SREBF1 and subsequent fatty acid metabolism-related genes were examined using qPCR assays in PTO1 with BHLHE40 knockdown and controls. (I) Protein levels of indicated genes in MIA PaCa-2 cells with BHLHE40 overexpression and controls. Data presentation: NES (normalized enrichment score, corrected for multiple comparisons using FDR method) and *P* value were showed in plots of (C); (G) data were the mean ± s.d. of n = 5 independent experiments; (H) data were the mean ± s.d. of n = 3 independent experiments. Statistical analysis: unpaired two-sided t-test for (G) and (H); $^*P<0.05$, $^{**}P<0.01$, $^{***}P<0.001$.

Figure S6. BHLHE40-mediated SREBF1 transcription activation facilitates PCa proliferation. (A)–(B) Results of CCK-8 assays were performed to evaluate the proliferation ability of PANC-1 and BxPC-3 cells with BHLHE40 knockdown. (C)–(F) Results of CCK-8 assay (C–D) and colony formation (E–F) were performed in PANC-1-shBHLHE40 and BxPC-3-shBHLHE40 cells with or without SREBF1 overexpression. Data presentation: (A), (B), (C) and (D) data were the mean ± s.d. of n = 6 independent experiments; (E) and (F) data were the mean ± s.d. of n = 3 independent experiments. Statistical analysis: two-way ANOVA for (A), (B), (C) and (D); unpaired two-sided t-test for (E) and (F); *P <0.05, **P <0.01, ***P <0.001.

Figure S7. BHLHE40 knockdown changes the global chromatin accessibility. (A) ATAC-seq summary plot of peak intensities from 5 kb upstream of transcription starting sites (TSS) to 5 kb downstream of transcription ending sites (TES) in PANC-1 cells with or without BHLHE40 knockdown. (B) Heatmap representation of normalized ATAC-seq signal in PANC-1 cells with or without BHLHE40 knockdown. Top panel shows read signals over the 938 hyper-accessible regions in PANC-1 BHLHE40 sh#1 cells, while the bottom panel shows read signals over the 2,423 hypo-accessible regions. Signals within 1.5 kb surrounding the center of DARs are displayed in descending order. (C) Bubble plot showing GO enrichment analysis of hypo-accessible region after BHLHE40 knockdown. (D) ChIP-seq summary plot of H3K27ac-binding intensities from 2 kb upstream of TSS to 2 kb downstream of TES in PANC-1 cells with or without BHLHE40 knockdown. (E) Set-up of SREBF1 enhancer-deleted PANC-1 cell line. Left three lanes: validation of CRISPR/Cas9-mediated deletion of the super-enhancer SREBF-E in PANC-1 cells with three independent replicates. Right two lanes: control of CRISPR/Cas9-mediated deletion in PANC-1 cells and ddH_2O. (F) Using ChIP-qPCR experiments, weaker enrichment of BRD4, MED1, Pol II, and H3K27ac in the enhancer region of SREBF1 was found in BHLHE40 knockdown compared with control PANC-1 cells. bp, base pair; ‘B sh’ or ‘BHLHE40 sh’, short for BHLHE40 short hairpin RNA. Data presentation: NES

(normalized enrichment score, corrected for multiple comparisons using FDR method) and *P* value were showed in plots of (C); (F) data were the mean ± s.d. of n = 3 independent experiments. Statistical analysis: unpaired two-sided t-test for (F); *P <0.05, **P <0.01, ***P <0.001.

Figure S8. H3K27ac and chromatin accessibility changes of BHLHE40 targets after BHLHE40 knockdown. (A–C) Multiple tracks in the IGV diagram exhibited co-occupancy of BHLHE40 and H3K27ac at the LYNX1, DBP, and HDAC5 promoter regions.

Figure S9. Phase-separation of BHLHE40 depends on its IDR regions. (A) Purified mEGFP, mEGFP-BHLHE40 (full length), mEGFP-BHLHE40 (177–319aa), and mEGFP-BHLHE40 (Δ177–319aa) were confirmed using Coomassie blue staining (M: marker and S: sample). (B) Droplet fusion ability of purified BHLHE40 with or without the loss of IDR region. Scale bar, 10 μm. (C) Expressions of SREBF1 protein levels were determined in cells with or without BHLHE40 knockdown, BHLHE40, and BHLHE40 mutant Δ(177–319) overexpression.

Figure S10. BHLHE40 knockdown induced apoptosis and ferroptosis in PCa. (A) Apoptosis was analyzed in the BHLHE40 of PANC-1 and BxPC-3 cells after BHLHE40 knockdown. (B–C) BHLHE40-silenced cells were treated with Necrostatin1 (10 μmol/L) or Z-VAD-FMK (10 μmol/L) for 48 h in PANC-1 and BxPC-3 cells, and cell viability was evaluated. (D)–(E) MDA concentration was detected before or after knockdown of SREBF1 in PANC-1 (D) and BxPC-3 (E) cells with or without 2 μmol/L Ferrostatin-1 treatment. (F) Confocal imaging revealed the effect of SREBF1 on lipid peroxidation. (G) Cell viability was examined in PANC-1 or BxPC-3 BHLHE40 sh #1 cells overexpressing SREBF1. Data presentation: (B), (C), (D), (E) and (G) data were the mean ± s.d. of n = 6 independent experiments; (A) and (F) data were the mean ± s.d. of n = 3 independent experiments. Statistical analysis: unpaired two-sided t-test for (A), (B), (C), (D), (E), (F) and (G); $^*P < 0.05$, $^{**}P < 0.01$, $^{***}P < 0.001$.

Figure S11. Loss of BHLHE40 promotes ferroptosis in PCa via SREBF1 *in vitro*. (A)–(B) Gross specimen demonstration and monitoring of tumor volumes of *in vivo* assays showed antioxidant function of NAC in BHLHE40-silenced tumors. (C)–(D) TEM imaging was conducted in PANC-1 cell-derived subcutaneous tumors or pancreatic tumoral organoids (PTO1 and PTO2) with or without BHLHE40 knockdown. Red arrows mark the shrunken mitochondria, and blue arrows mark the lipid droplets. Scale bar, 2 μm. (E) Quantification of shrunken mitochondria and lipid droplets for TEM experiments. T, subcutaneous tumor samples. (F)–(H) Cell viability (F), MDA concentration (G), and BODIPY 581/591 C11 detection (H) in PTO1 or PTO2 treated with ferrostation-1 (2 μmol/L) for 72 h after BHLHE40 knockdown. (I)–(K) Cell viability (I), MDA concentration (J), and BODIPY 581/591 C11 detection (K) in PTO1 or PTO2 BHLHE40 sh #1 overexpressing SREBF1. Data presentation: (F), (G), (I) and (J) data were the mean ± s.d. of n = 6 independent experiments; (B) data were the mean ± s.d. of n = 5 independent experiments (H) and (K) data were the mean ± s.d. of n = 3 independent experiments. Statistical analysis: unpaired two-sided t-test for (F), (G), (H), (I), (J) and (K); two-way ANOVA for (B); *$P < 0.05$, **$P < 0.01$, ***$P < 0.001$.
