# Supplementary material for: BHLHE40 Inhibits Ferroptosis in Pancreatic Cancer Cells via Upregulating SREBF1
Source: Adv Sci (Weinh). 2023 Dec 8;11(7):2306298. doi: 10.1002/advs.202306298 (PMC10870036; doi:10.1002/advs.202306298)

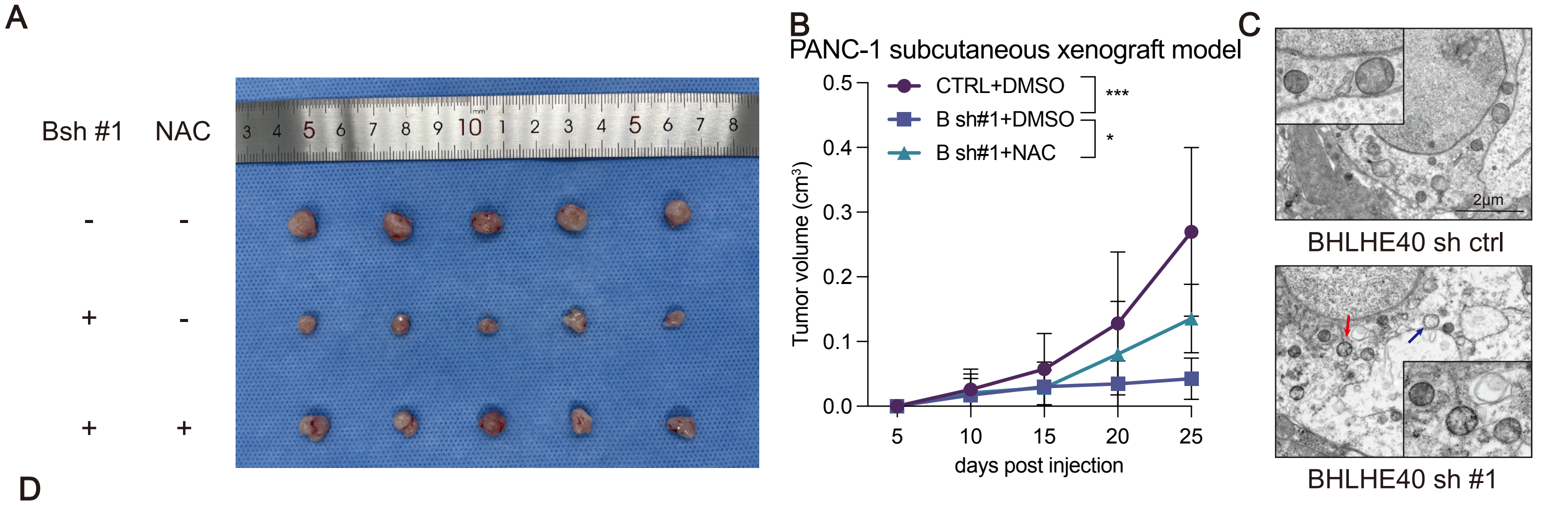

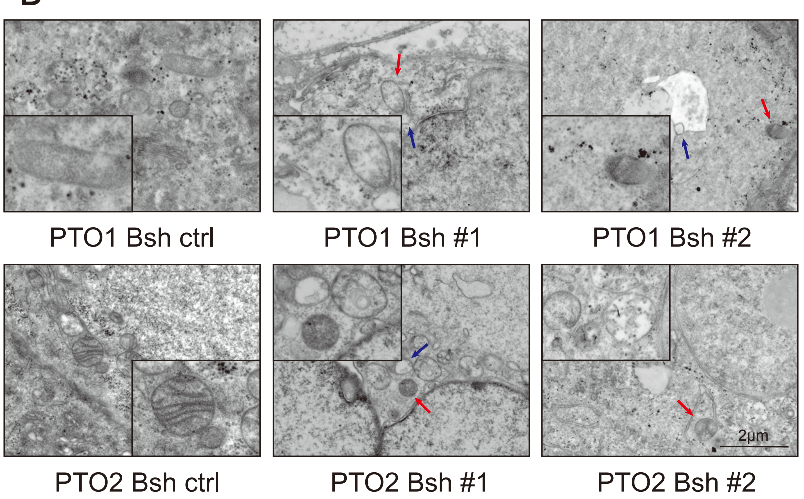

E

Items/ Grades	Number of Damaged Mitochondria			Number of Lipid Droplets		
	1	2	3	1	2	3
Cell Control	9	8	4	0	0	0
Cell Bsh #1	11	19	10	1	3	1
Cell Ssh #1	17	18	13	2	2	1
Cell Erastin	25	24	17	6	5	4
T Bsh ctrl	7	11	5	1	1	1
T Bsh #1	19	24	11	2	3	2
PTO1 Bsh ctrl	8	9	8	0	2	10
PTO1 Bsh #1	19	15	32	2	2	5
PTO1 Bsh #2	22	20	22	3	9	14
PTO2 Bsh ctrl	5	12	6	3	0	0
PTO2 Bsh #1	11	23	31	0	6	0
PTO2 Bsh #2	13	20	14	9	4	1

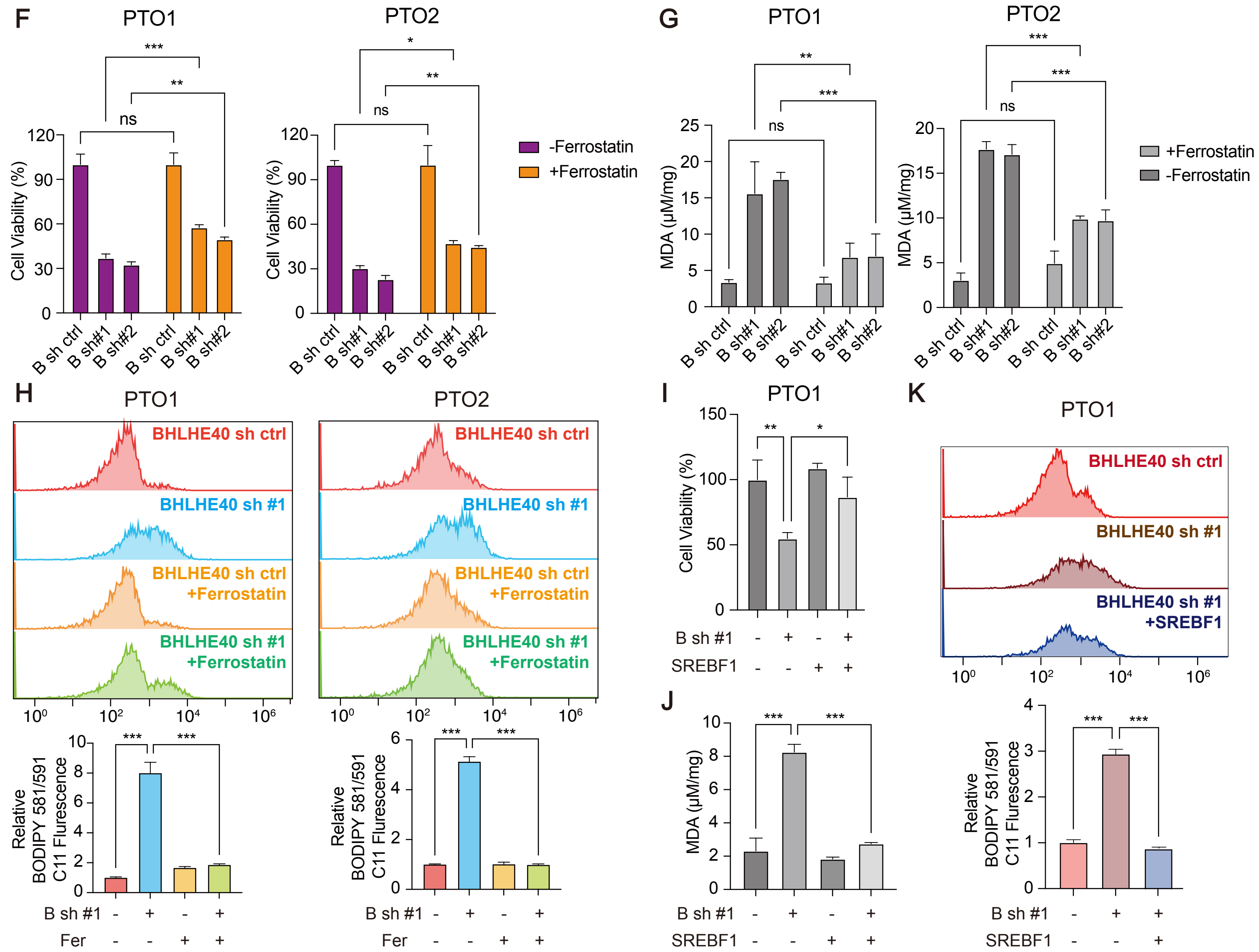

Supplement: Supplementary file 5 — Supplemental Figure [file ADVS-11-2306298-s005.zip › Figure S11.pdf]
