# Supplementary figures and images for: BHLHE40 Inhibits Ferroptosis in Pancreatic Cancer Cells via Upregulating SREBF1
Source: Adv Sci (Weinh). 2023 Dec 8;11(7):2306298. doi: 10.1002/advs.202306298 (PMC10870036; doi:10.1002/advs.202306298)

A

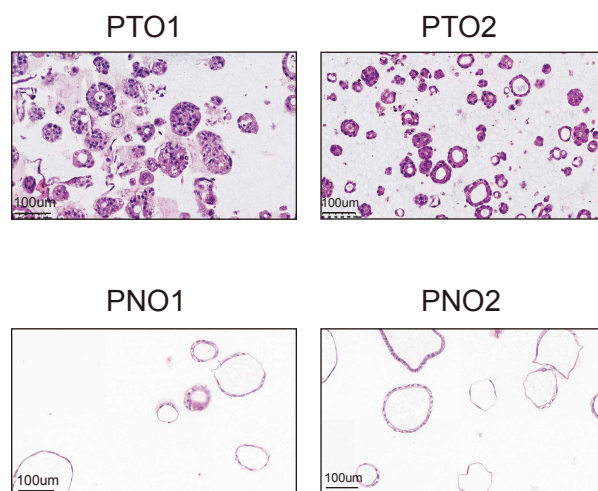

B

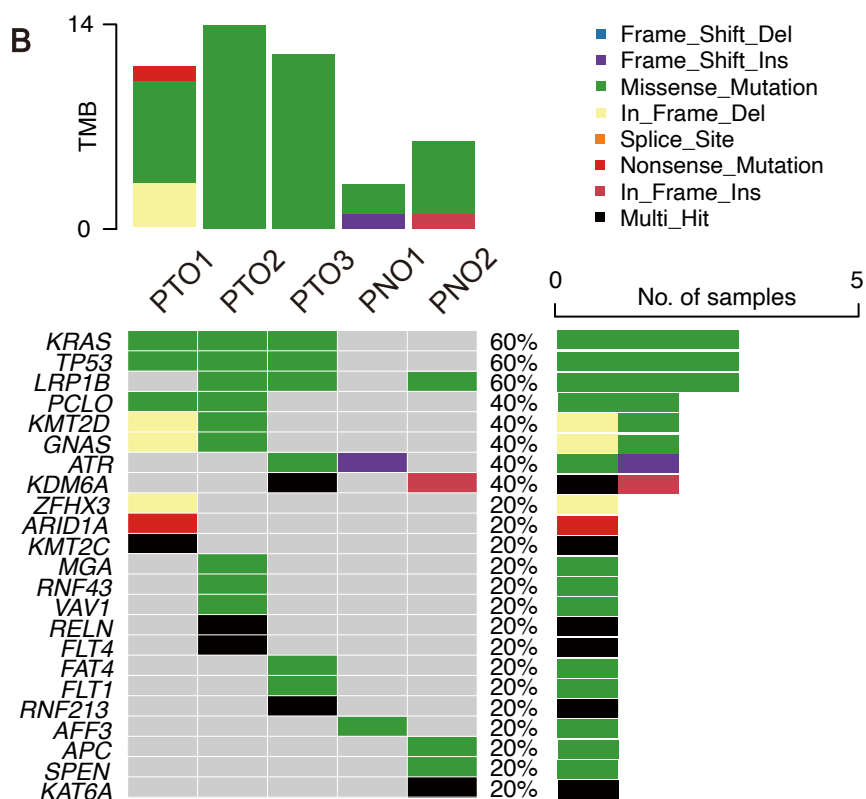

C

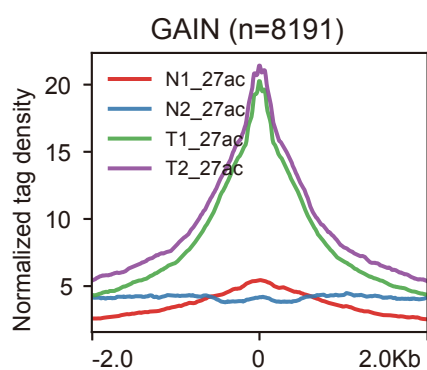

D

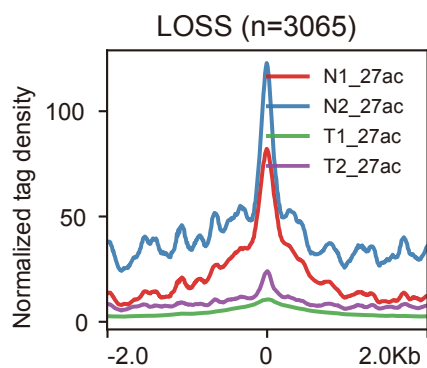

E

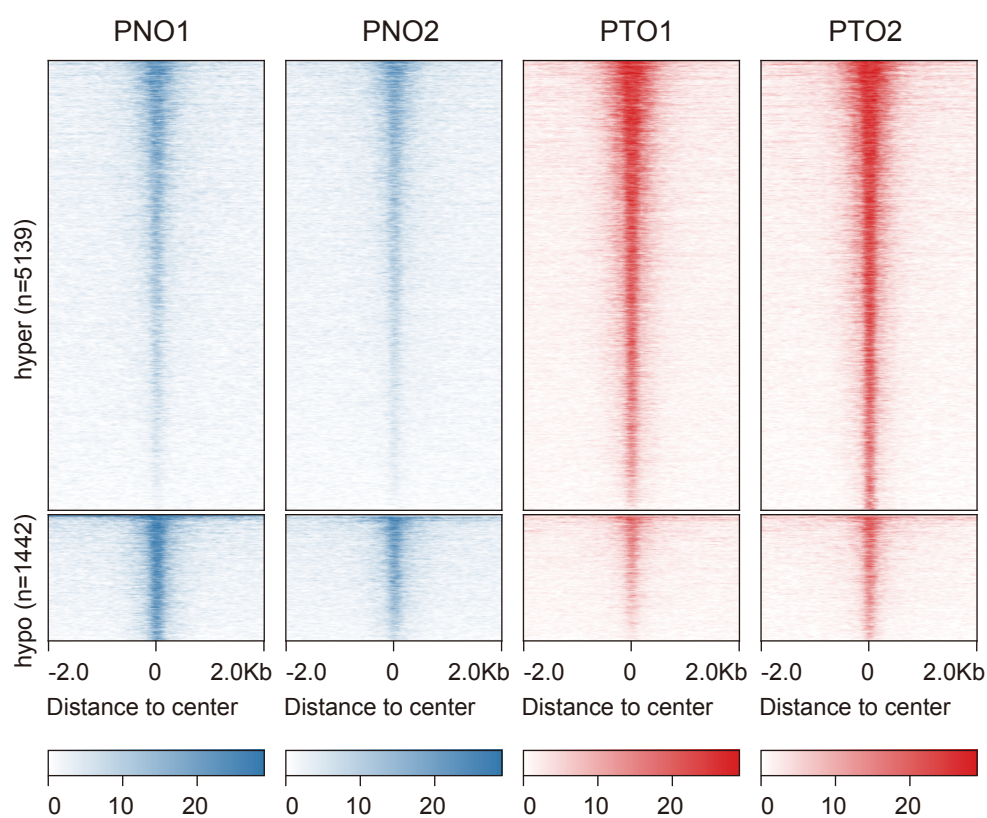

Supplement: Supplementary file 5 — Supplemental Figure [file ADVS-11-2306298-s005.zip › Figure S1.pdf]

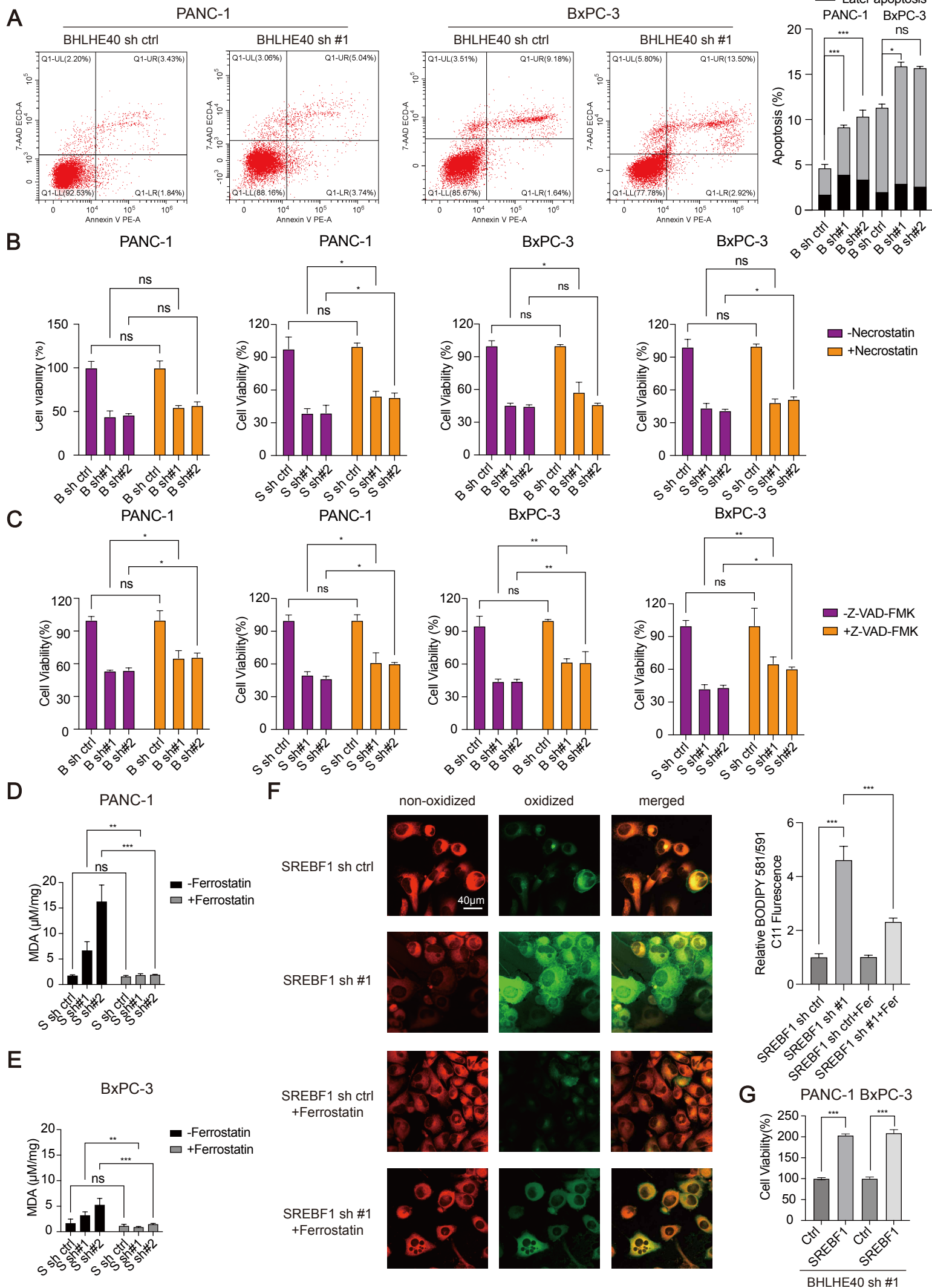

Supplement: Supplementary file 5 — Supplemental Figure [file ADVS-11-2306298-s005.zip › Figure S10.pdf]

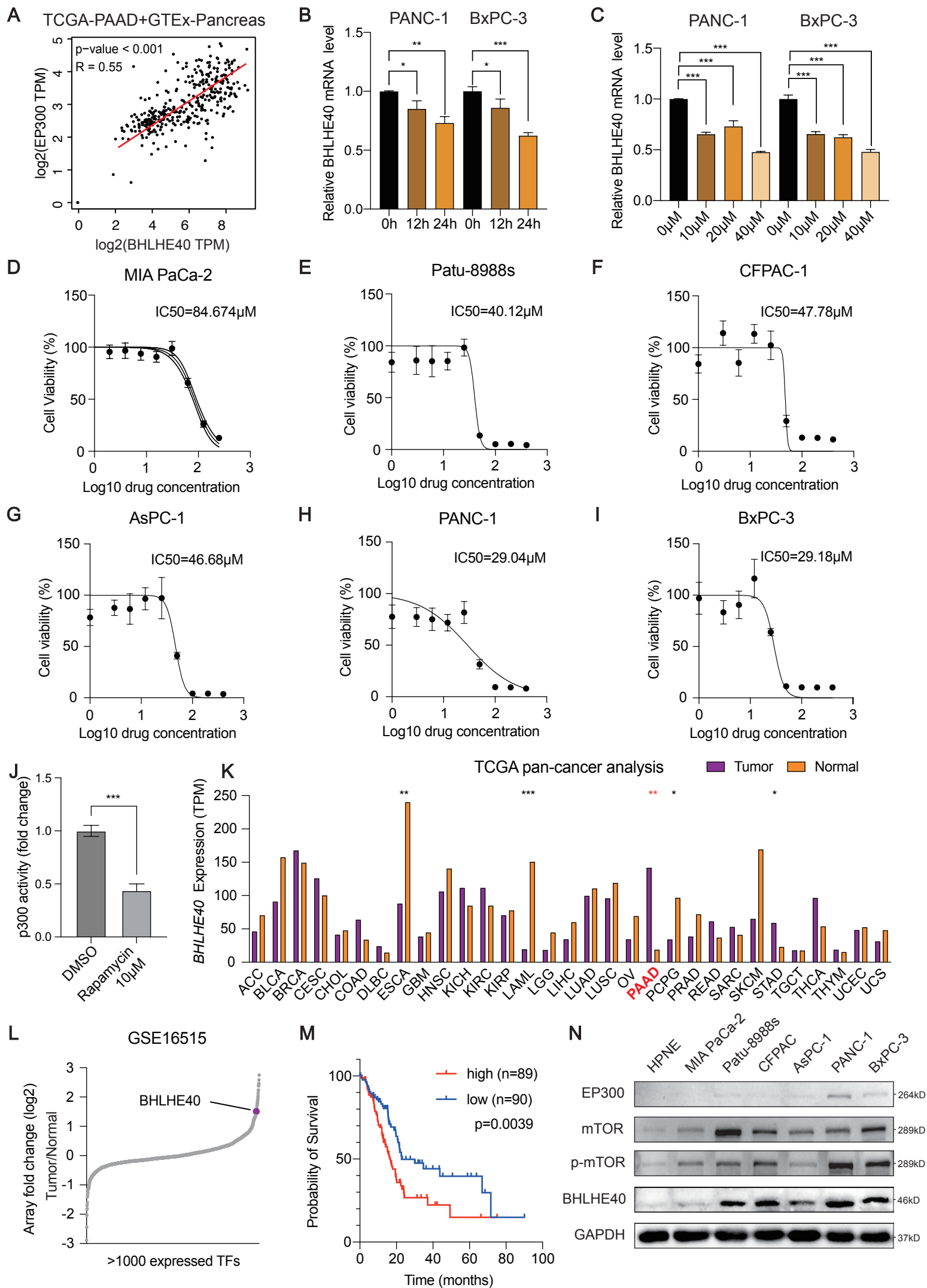

Supplement: Supplementary file 5 — Supplemental Figure [file ADVS-11-2306298-s005.zip › Figure S2.pdf]

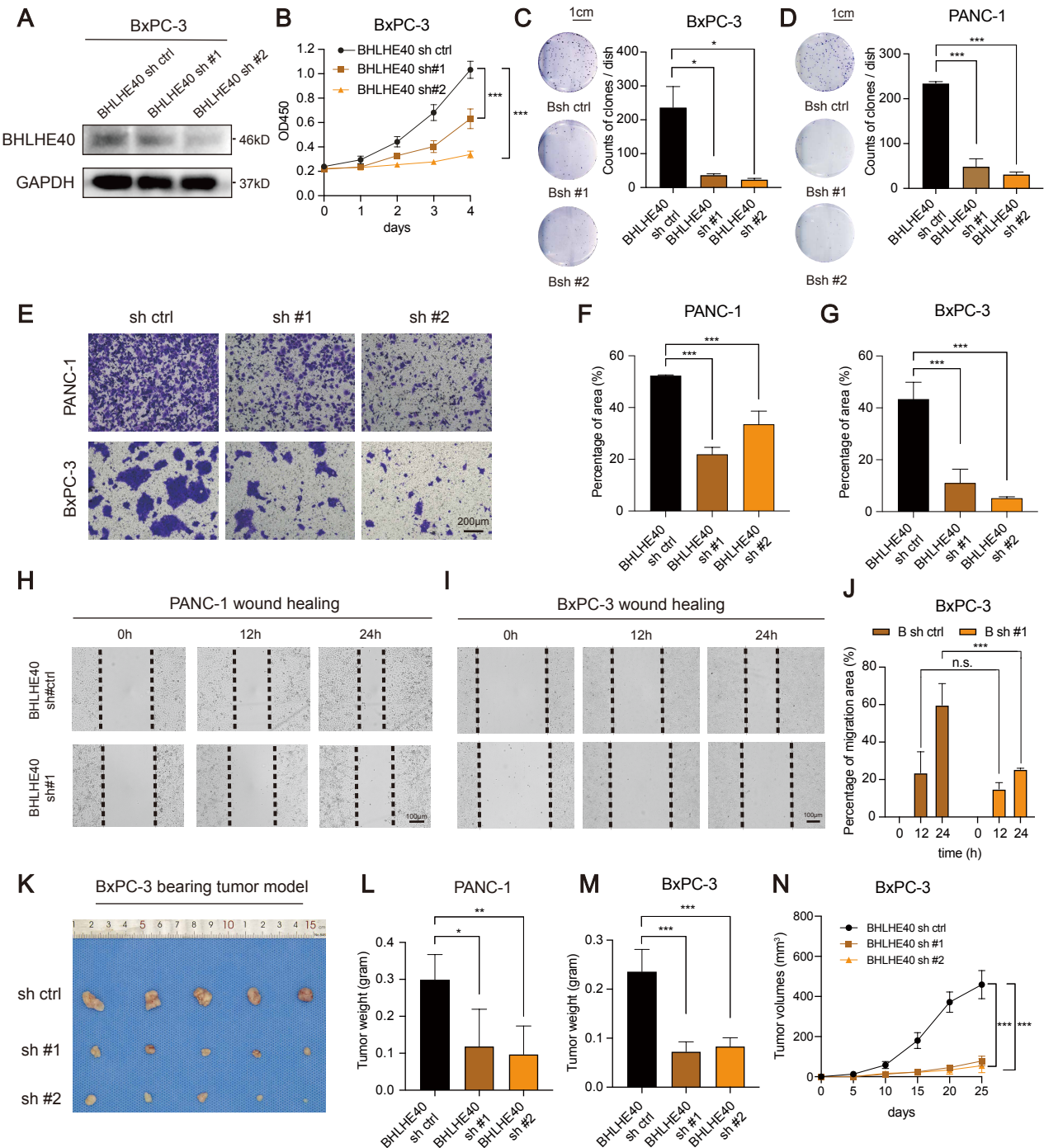

Supplement: Supplementary file 5 — Supplemental Figure [file ADVS-11-2306298-s005.zip › Figure S3.pdf]

**A**

BHLHE40 sh ctrl

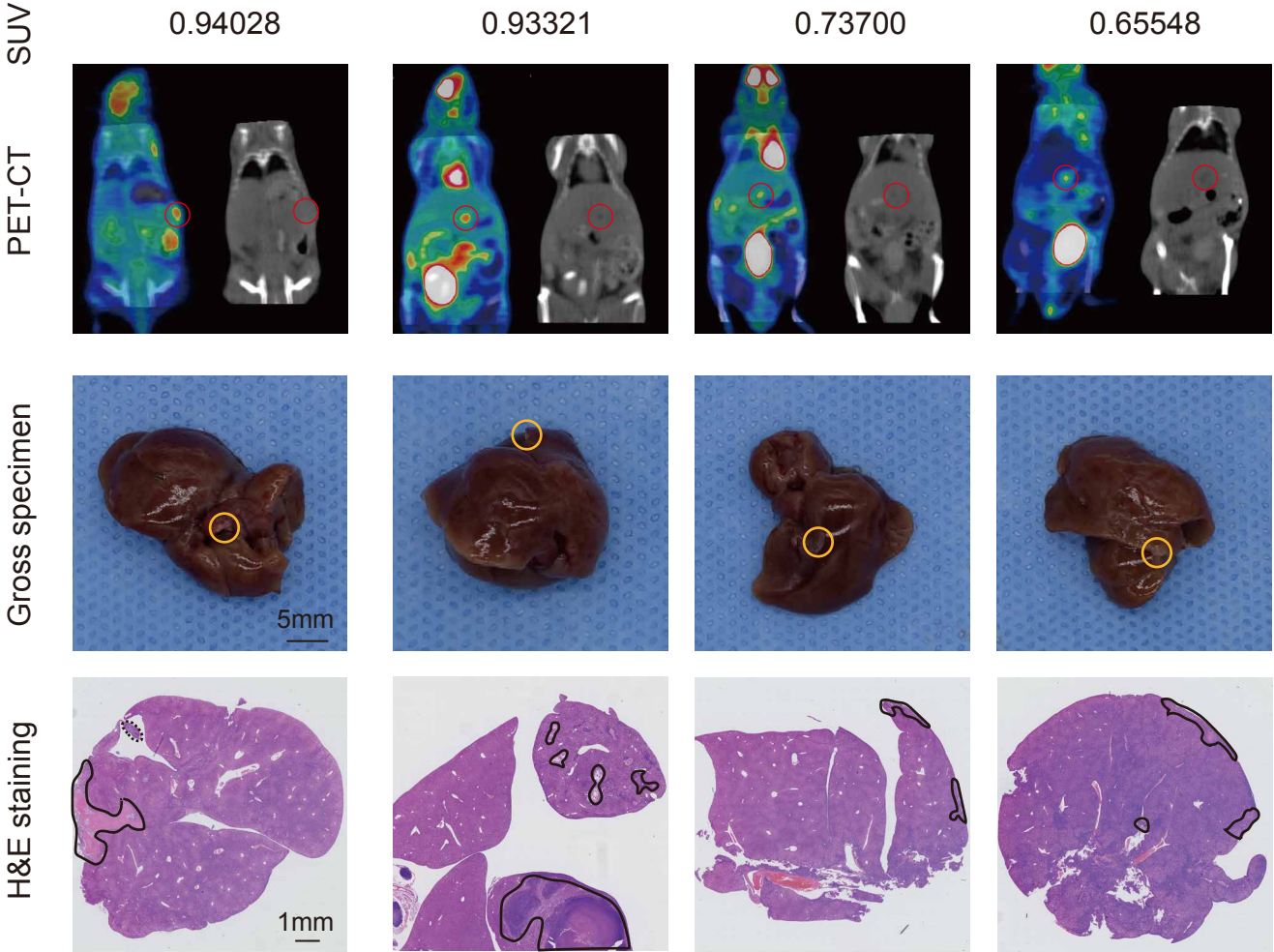

**B**

BHLHE40 sh #1

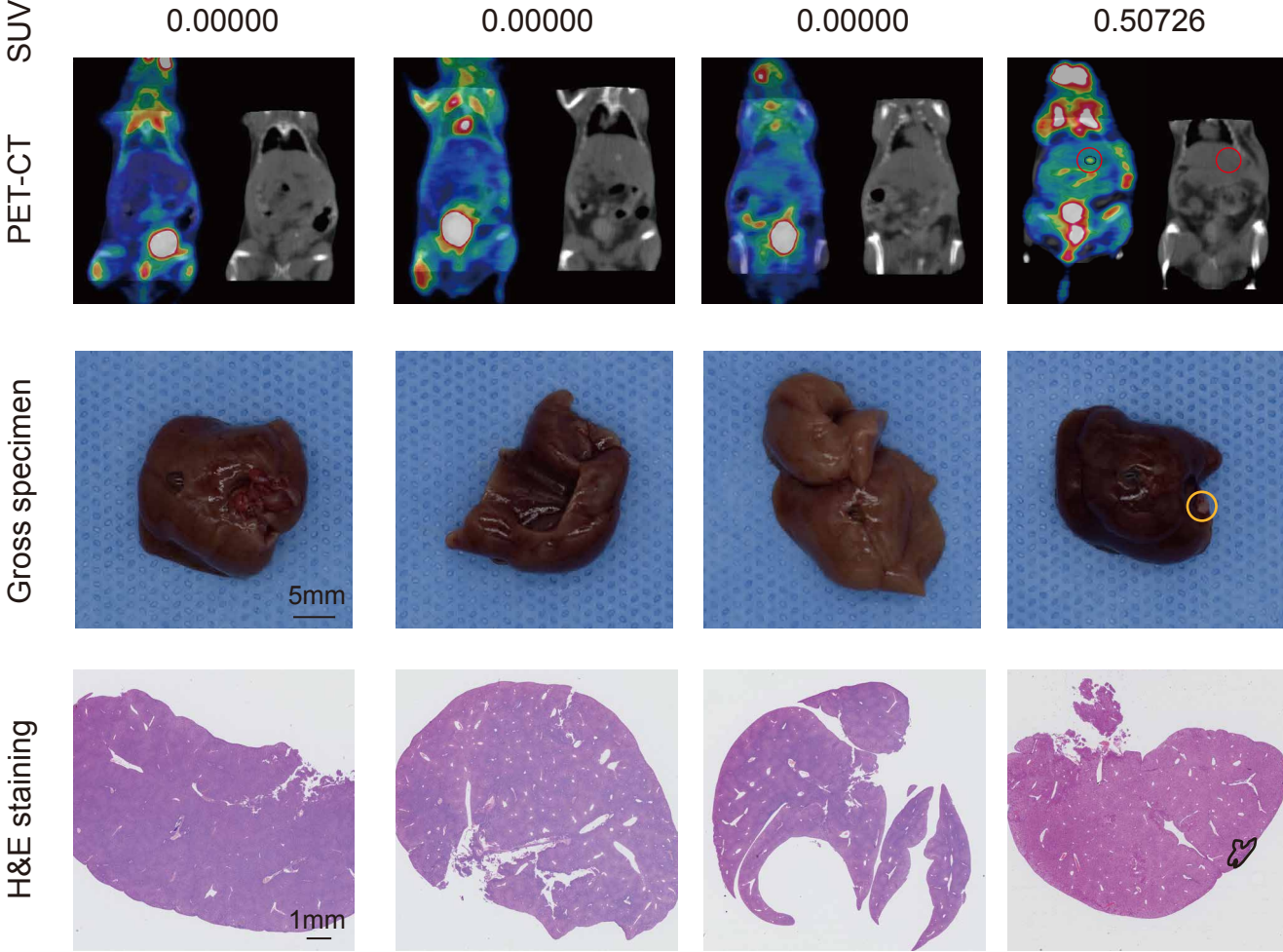

Supplement: Supplementary file 5 — Supplemental Figure [file ADVS-11-2306298-s005.zip › Figure S4.pdf]

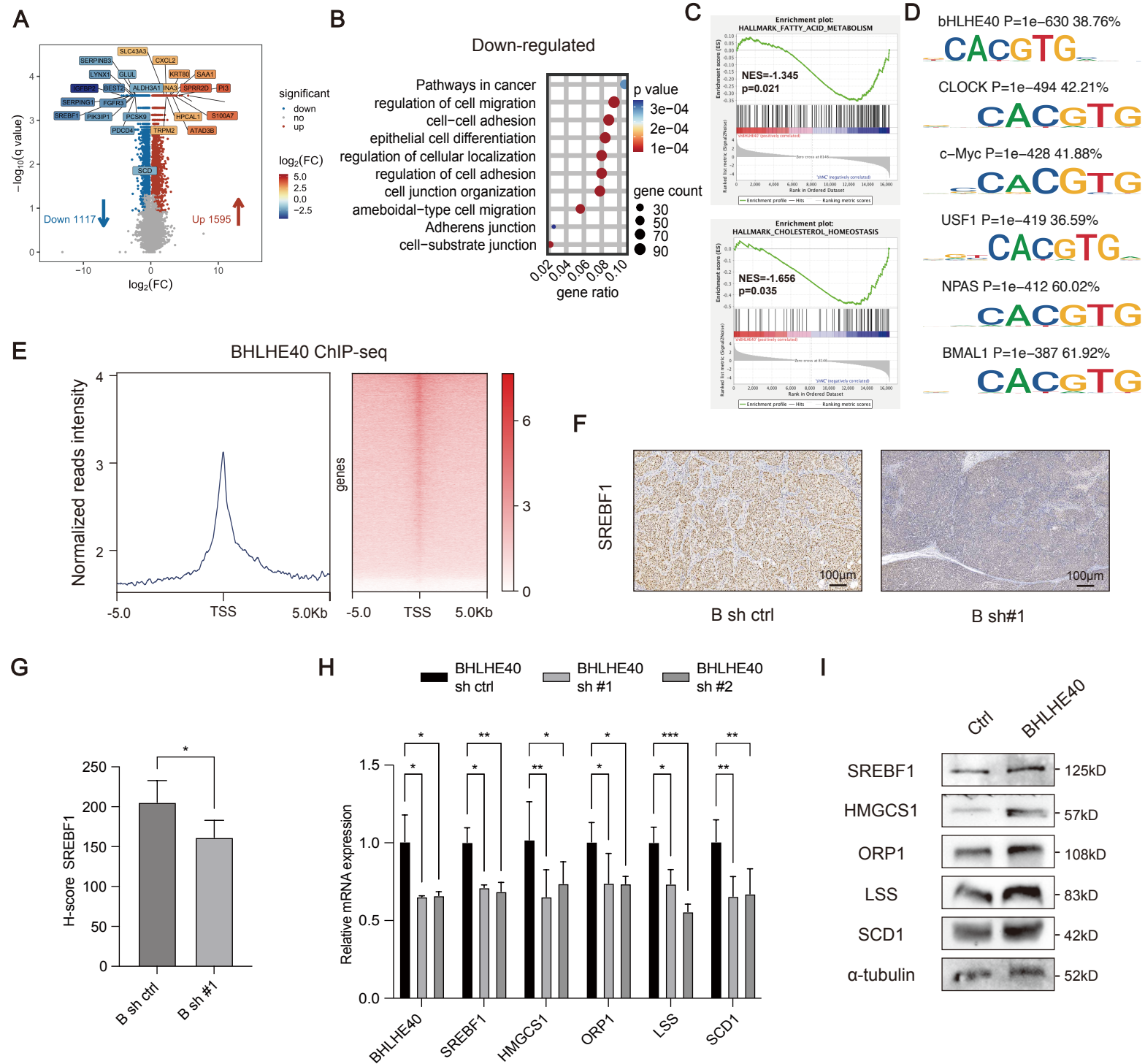

Supplement: Supplementary file 5 — Supplemental Figure [file ADVS-11-2306298-s005.zip › Figure S5.pdf]

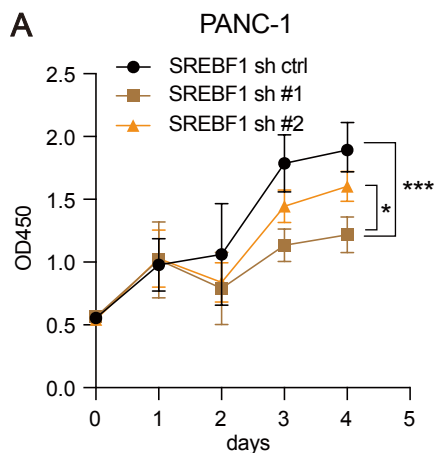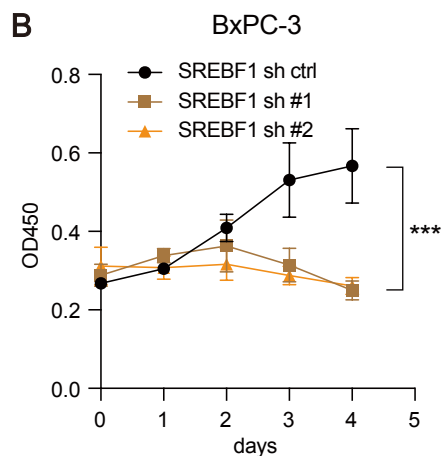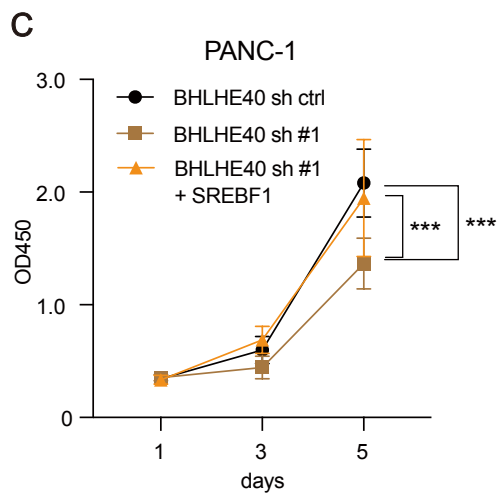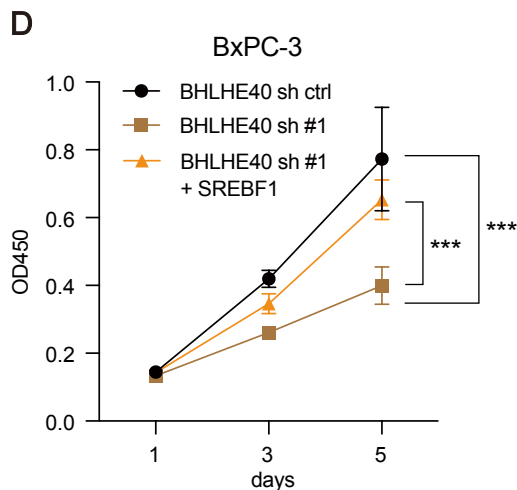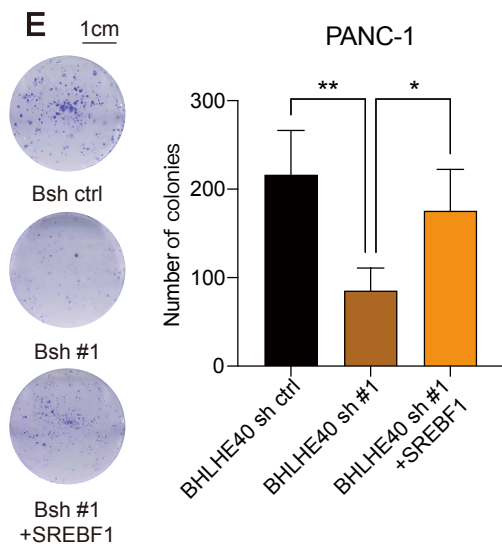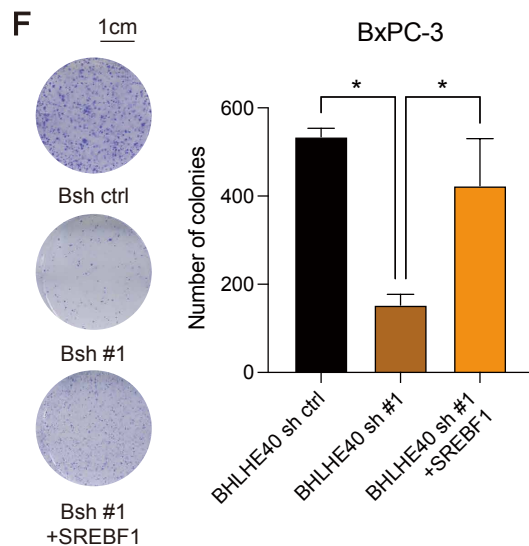

Supplement: Supplementary file 5 — Supplemental Figure [file ADVS-11-2306298-s005.zip › Figure S6.pdf]

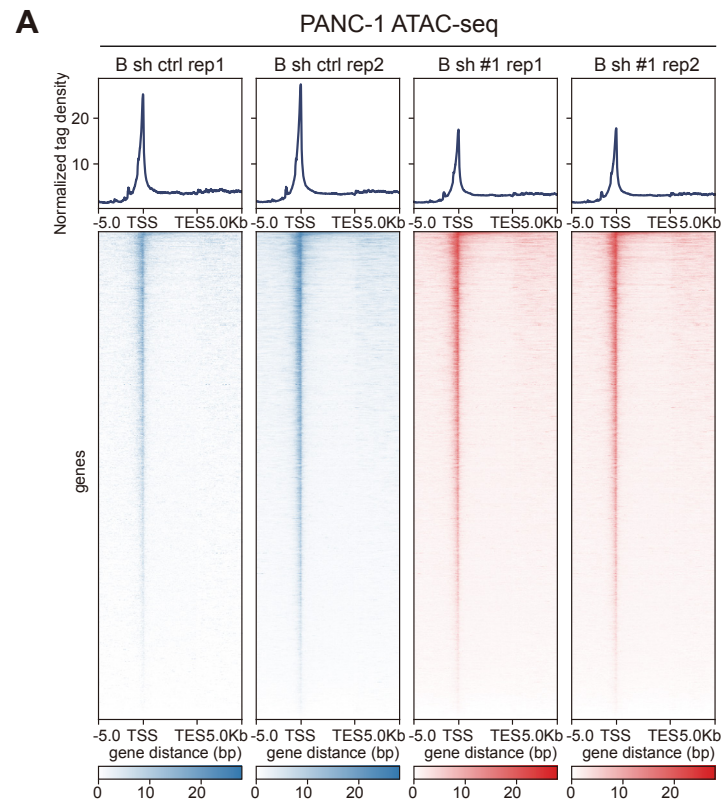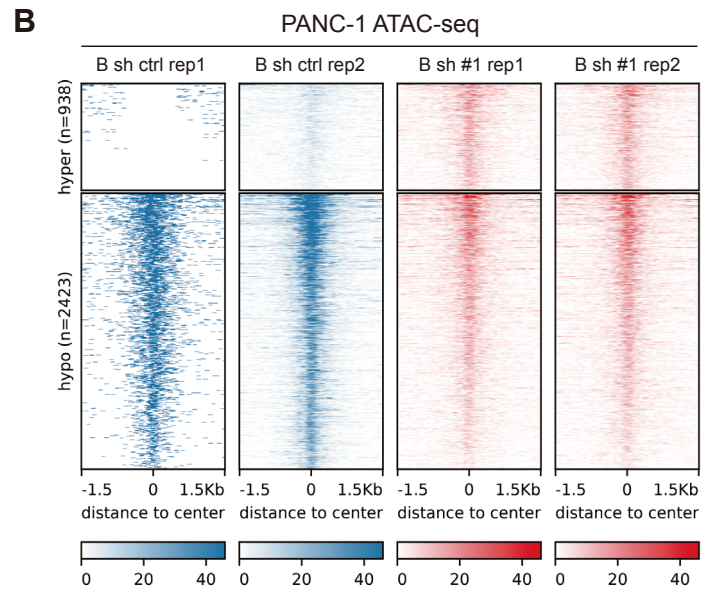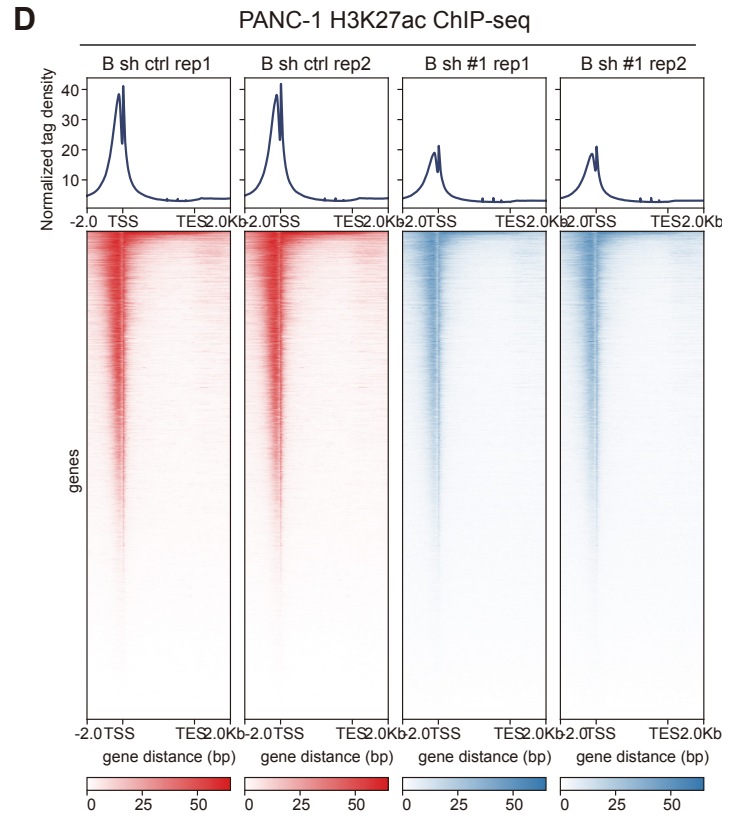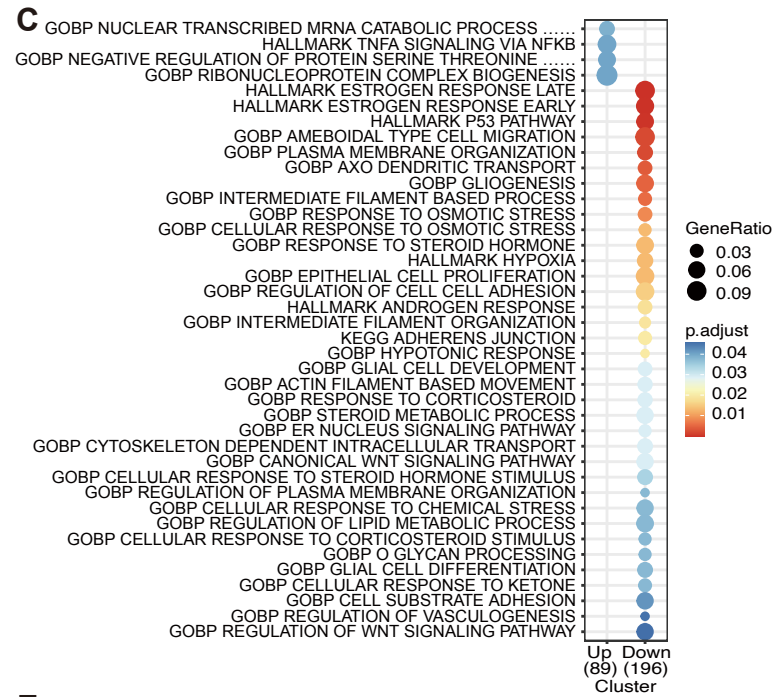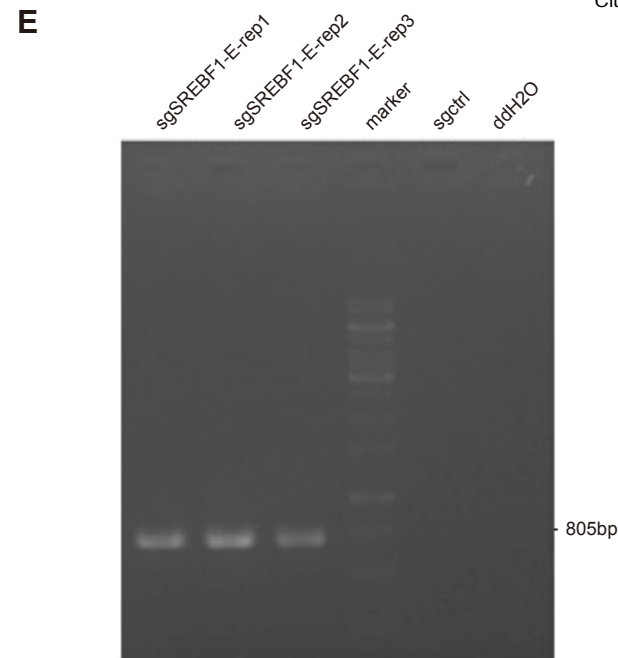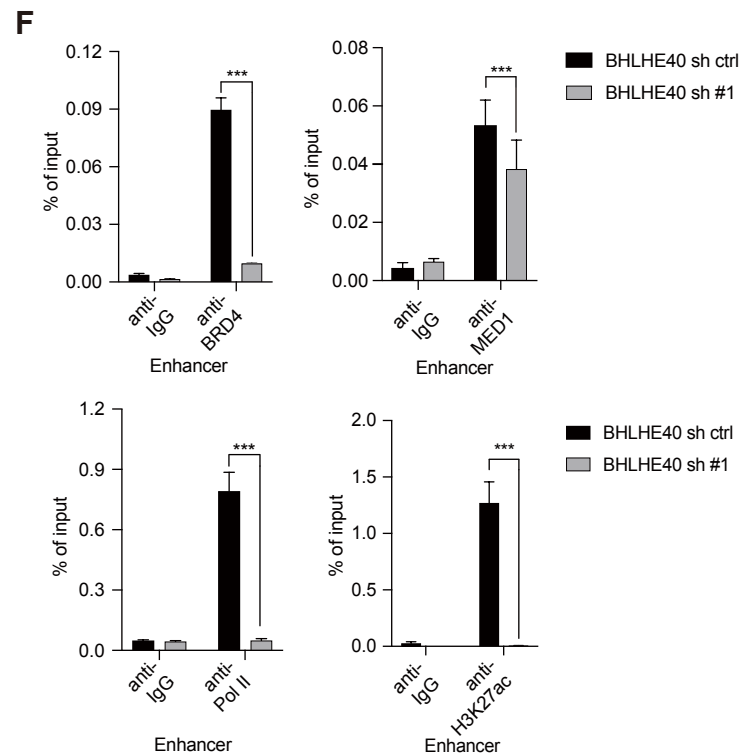

Supplement: Supplementary file 5 — Supplemental Figure [file ADVS-11-2306298-s005.zip › Figure S7.pdf]

**A***chr8:143839996-143871057*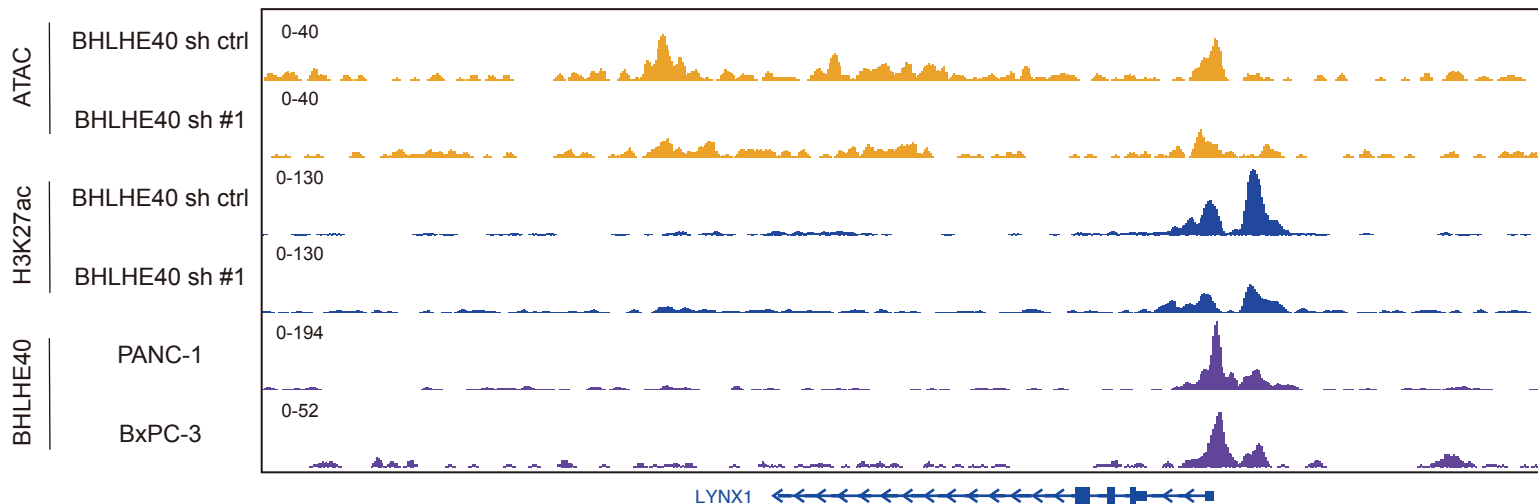**B***chr19:49131099-49143526*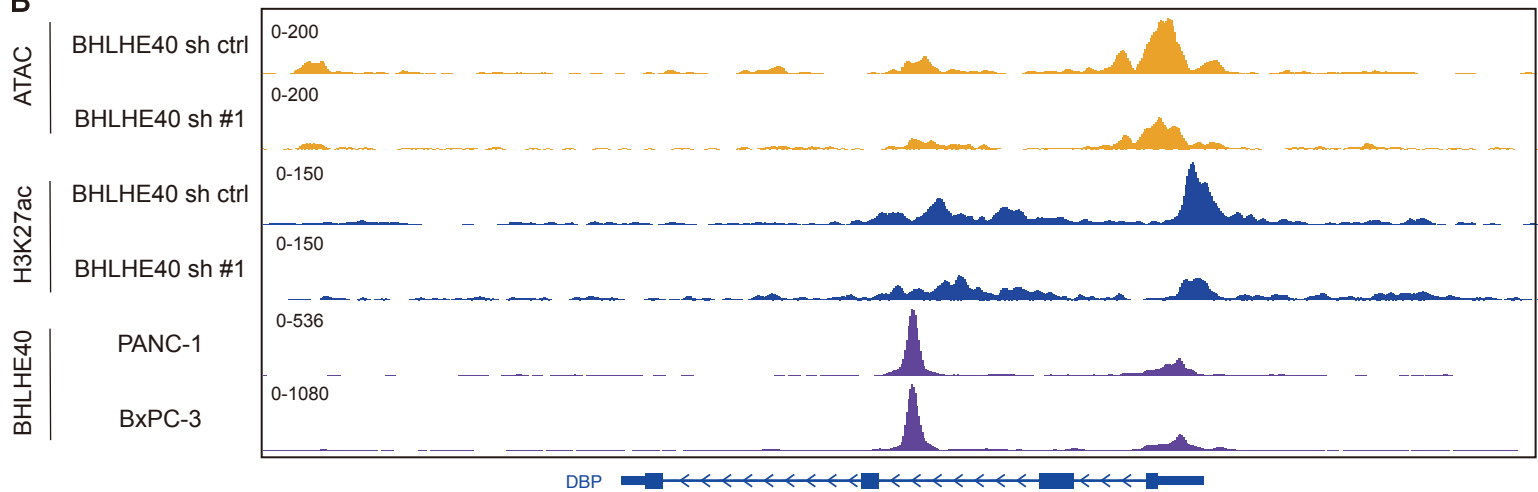**C***chr17:42146305-42208830*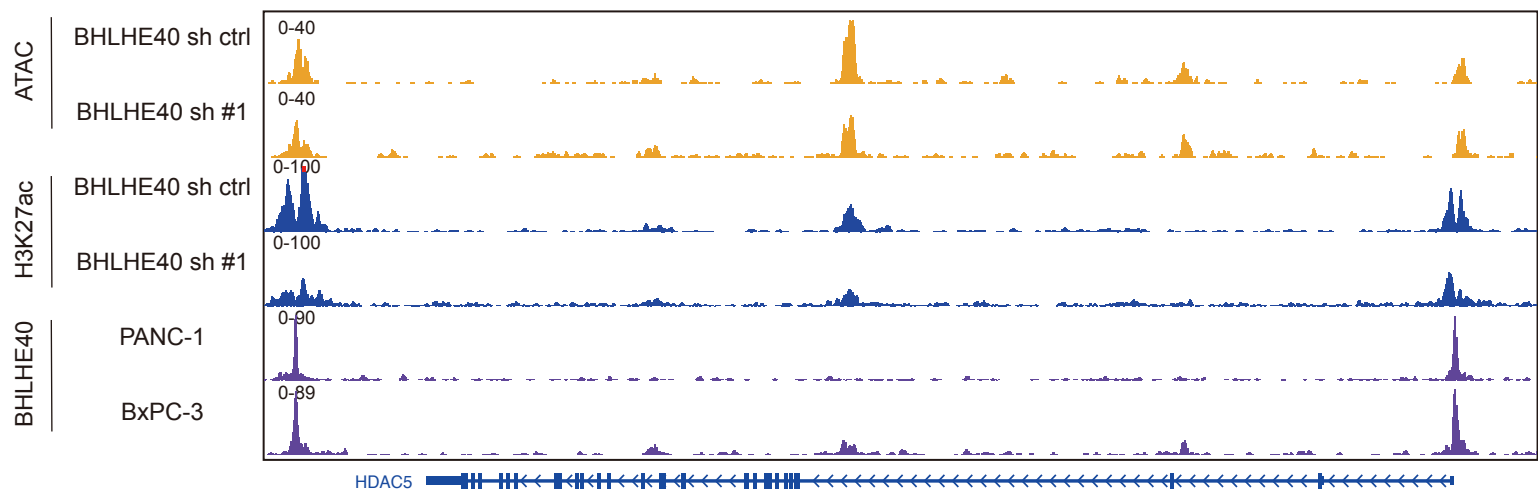

Supplement: Supplementary file 5 — Supplemental Figure [file ADVS-11-2306298-s005.zip › Figure S8.pdf]

**A**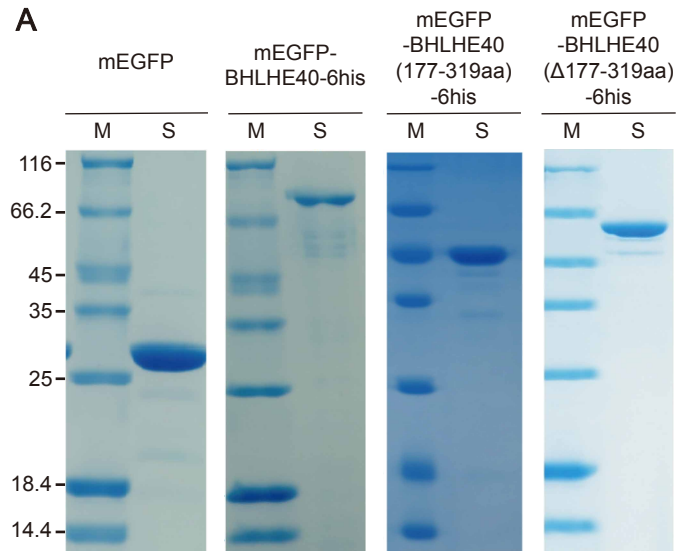**B**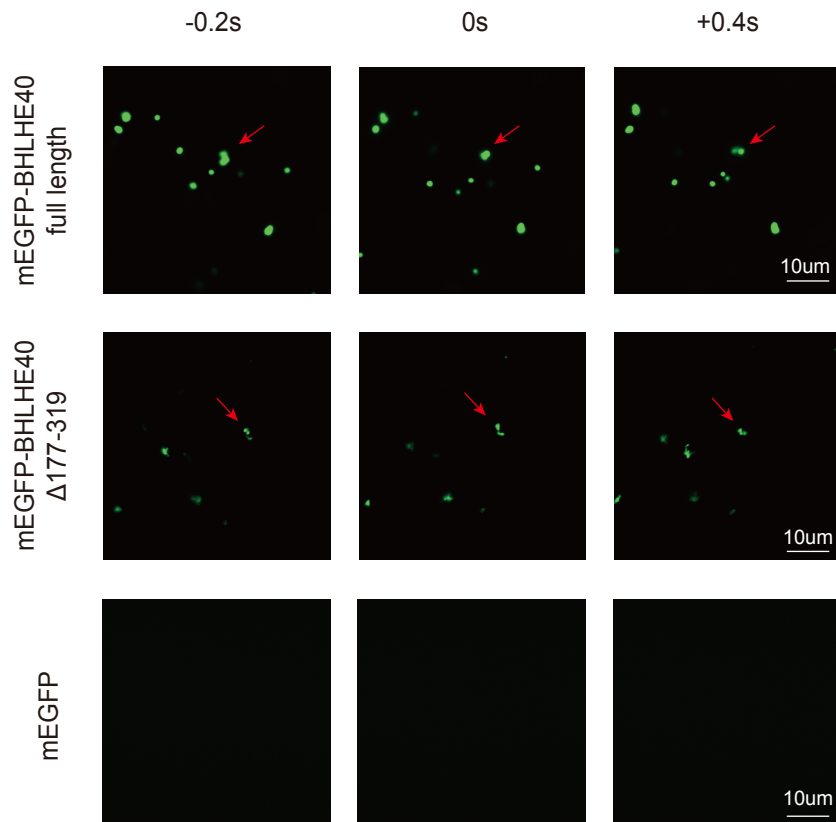**C**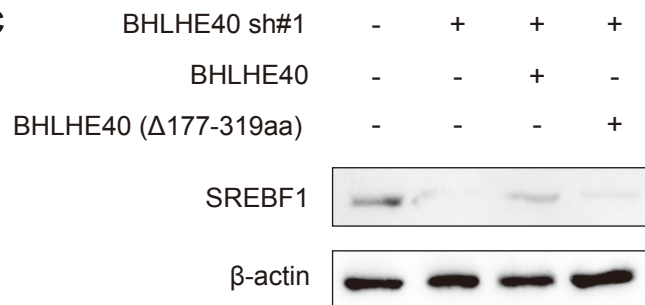

Supplement: Supplementary file 5 — Supplemental Figure [file ADVS-11-2306298-s005.zip › Figure S9.pdf]
